# Analysis and construction of the coal and rock cutting state identification system in coal mine intelligent mining

**DOI:** 10.1038/s41598-023-30617-9

**Published:** 2023-03-01

**Authors:** Meichen Zhang, Lijuan Zhao, Baisheng Shi

**Affiliations:** 1 College of Mechanical Engineering, Changshu Institute of Technology, Suzhou, 215500 China; 2 College of Mechanical Engineering, Liaoning Technical University, Fuxin, 123000 China; 3 The State Key Lab of Mining Machinery Engineering of Coal Industry, Liaoning Technical University, Fuxin, China; 4 Liaoning Province Large Scale Industrial and Mining Equipment Key Laboratory, Fuxin City, 123000 Liaoning Province People’s Republic of China

**Keywords:** Energy science and technology, Engineering

## Abstract

The recognition of cutting state of coal-rock is the key technology to realize “unmanned” mining in coal face. In order to realized real-time perception and accurate judgment of coal-rock cutting state information, this paper combined the field test sampling, construction technology of complex coal seam, virtual prototype technology, bidirectional coupling technology, data processing theory, image fusion method, and deep learning theory to carry out multi domain deep fusion experimental research on multi-source heterogeneous data of coal and rock cutting state. The typical complex coal seam containing gangue, inclusion, and minor fault in Yangcun mine of Yanzhou mining area was taken as the engineering object. The high-precision three-dimensional simulation model of the complex coal seam that can update and replace particles was constructed. Based on the simulation results of Discrete Element Method-Multi Flexible Body Dynamics (DEM-MFBD), the one-dimensional original vibration acceleration signals of the key components of the shearer cutting part were determined, including spiral drum, rocker arm shell, and square head. After transforming one-dimensional original signal data into two-dimensional time–frequency images by Short-time Fourier Transform, morphological wavelet image fusion technology was used to realize the effective fusion of characteristic information of spiral drum, rocker arm shell, and square head under different working conditions. Based on the deep learning theory, the DCGAN-RFCNN (Deep Convolutional Generative Adversarial Networks-Random Forest Convolutional Neural Networks) coal and rock cutting state recognition network model was constructed. Combining convolution neural network with random forest recognition classifier, RFCNN coal and rock cutting state recognition classification model was constructed, and the recognition network model was trained to obtain the model recognition results. Through the comparative experimental analysis of the RFCNN network model with different recognition network models and different synthetic sample numbers in the recognition network, the effectiveness of the recognition network model was verified. The results show that: When synthetic samples are not included in each working condition in the RFCNN model, the average recognition rate is 90.641%. With the increase of the number of synthetic samples, the recognition rate of coal and rock cutting state increases. When the number of synthetic samples added to each working condition reaches 5000, the recognition effect is the best, and the average recognition rate reaches 98.344%, which verifies the superiority of enriching the data set by using the improved DCGAN network. Also, the RFCNN outperformed the other variants: it obtained higher recognition accuracy by 25.085, 21.925 and 19.337%, respectively, over SVW, CNN, and AlexNet. Also, the experimental platform of shearer cutting coal and rock was built, where the coal and rock cutting state recognition network was trained and tested based on the migration learning theory. Through the statistical test results, the accuracy of coal and rock cutting state recognition is 98.64%, which realizes the accurate recognition of coal and rock cutting state.

## Introduction

90% of China’s coal is mined by underground mining, and the intelligent level of mining equipment is low, which leads to many coal mining disasters, weak adaptability of coal machinery, high failure rate, and low efficiency. Improving the intelligent level of coal machinery equipment is one of the main tasks of coal mine intelligent development^1–5^. Shearer is the core equipment of a fully mechanized mining face. The accurate identification of its cutting state is not only the key to realizing the intelligent and efficient cutting of shearer, but also the necessary basic guarantee of intelligent unmanned mining in a fully mechanized mining face. Many scholars have carried out research in this area. Ankita Singh et al.^6^ combined with the gray information of coal and rock, selected the grayscale threshold to segment the coal and rock image, and designed the Gray Level Co-occurrence Matrix to extract the features of the segmented image, so as to achieve the purpose of identifying coal and rock with different properties. Sushma Kumari et al.^7^ realized the depth perception of the cutting target of mining machinery through real-time image mosaic, image enhancement, CNN Network and other processing methods based on the intelligent vision enhancement technology, so as to achieve the purpose of intelligent mining of mining machinery under harsh conditions. Wang et al.^8^ processed the cutting force signal in the cutting process of shearer through DBC technology, and obtained the power spectrum, variance and other characteristics of the signal, so as to realize the identification of the coal and rock cutting state. Zhang et al.^9^ constructed a coal and rock cutting vibration signal recognition network based on cepstrum distance. The problem of low adaptability and sensitivity of conventional methods was solved through taking cepstrum distance as the eigenvalue of recognition network, so as to realize the judgment of coal and rock cutting state. Cheng et al.^10^ built a coal and rock mixed medium analysis test platform based on Bruggeman medium theory. The experimental results show that the coal content detection model can quantitatively describe the cutting state of coal and rock. Lu et al.^11^ extracted the vibration signal of auger bit of drilling shearer during operation. At the same time, the Wavelet decomposition was used to obtain the characteristic vector of the signal, and finally the coal and rock cutting state recognition model was successfully constructed based on BP Neural Network. Wang et al.^12^ mentioned in the latest development technology of coal mine intelligence that the coal and rock intelligent sensing technology based on multi-source data information is the key content of intelligent mining. Sun et al.^13^ constructed a new coal and rock recognition evaluating indicat based on the improved YOLOv3 depth perception intelligent recognition algorithm, so as to improve the accuracy of the coal and rock recognition.

The shearer working under the condition of coal and rock with gangue has bad working conditions and complex environment. The occurrence conditions of cut coal and rock, the kinematic parameters of shearer, the gradual change characteristics of power transmission system and the interaction between spiral drum and coal and rock will affect the cutting and crushing process of shearer directly or indirectly. Although the research on recognition technology based on coal and rock image can realize the recognition of coal and rock interface, the adverse underground environment makes it impossible to obtain coal and rock image with clear characteristics. At the same time, the complexity and diversity of coal and rock characteristics also restrict the recognition accuracy^14^. Although the research of Ground penetrating radar and other technologies^15^ is not affected by the underground mining environment, the recognition accuracy is low and the effect is poor due to long-distance transmission, so it is difficult to realize mining while detecting. Therefore, how to accurately and quickly perceive the cutting state of coal and rock online and then realize the real-time control of the attitude of the shearer spiral drum is still the technical bottleneck to realize the intelligent and efficient cutting of the shearer, which still needs to be deeply studied.

Based on this, we combined the field test sampling, construction technology of complex coal seam, virtual prototype technology^16–20^, bidirectional coupling technology, data processing theory, image fusion method, and the deep learning theory^21,22^ to carry out multi domain deep fusion experimental research on multi-source heterogeneous data of coal and rock cutting state. We constructed a high-precision 3D simulation model for a complex coal seam and completed the bidirectional coupling model between it and the cutting part of the shearer. Then, we obtained the vibration signal of the coal and rock cutting state. Reasonable data information conversion and fusion rules were designed, and the raw data information image set of coal and rock cutting state was constructed. A sample expansion method was constructed based on the analysis of the characteristics of the image dataset, and a coal and rock cutting state recognition network was designed combined with the image information. This provides a technical foundation and theoretical method for the successful application of coal and rock cutting state identification technology in the intelligent development of coal mines.

## Extraction and analysis of vibration signals in the coal and rock cutting

### Construction of high-precision 3D simulation model for complex coal seam

#### Construction of initial basic model for high-precision 3D simulation of complex coal seam

**Figure 1:**
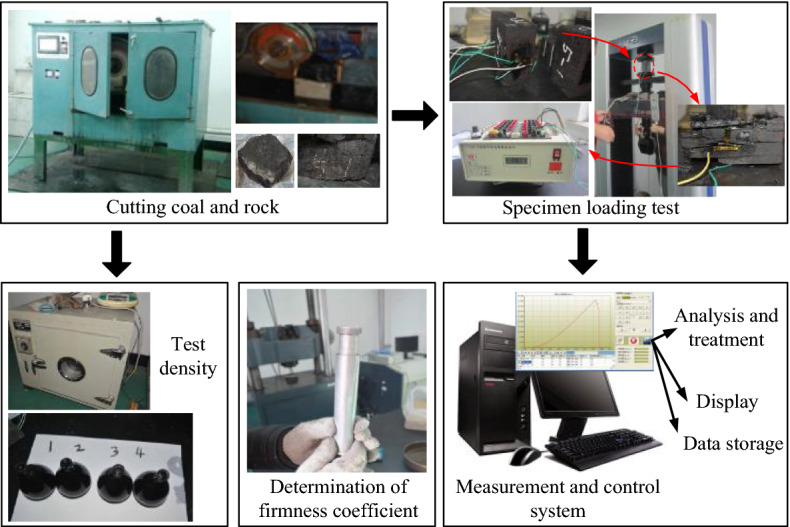
Tests of the physical and mechanical parameters of coal-rock.

The average thickness of of 17 layers in the Yanzhou mining area is 1 m. The dip angle of coal seam is 5° ~ 13°. The firmness coefficient of coal seam is 1.39, with wide distribution and stable occurrence conditions. However, the coal seam structure is complex and generally contains iron sulfide inclusion. The thickness and length of inclusions are generally 100 ~ 200 mm and 200 ~ 300 mm respectively. The firmness coefficient of inclusions reaches 8.4, and the distribution density is 0.88 pieces/m^2^^23,24^ The coal seam contains 1 ~ 2 layers of gangue, with a thickness of 0.02 ~ 0.44 m, and the lithology is carbon-rich sandstone. The roof of the coal seam is limestone, with an average thickness of 5.85 m. The floor of the coal seam is aluminum mudstone, with an average thickness of 1.17 m. The coal seams in Yanzhou mining area were sampled and tested according to the sampling principle and testing standards^25,26^. The relevant experiments are shown in Fig. 1. Table 1 shows the specific physical and mechanical properties parameters of coal and rock obtained from the experiment.

**Table 1:** Physical and mechanical property parameters of the coal and rock.

Coal and rock	Density (kg/m3)	Elastic modulus (MPa)	Poisson’s ratio	Tensile strength (MPa)	Compressive strength (MPa)	Soundness coefficient/*f*
Coal 1	1280	2010	0.28	0.3	12	1.4
Coal 2	1319	5240	0.31	1.73	23.79	2.38
Coal 3	1420	9560	0.15	2.31	34.26	3.8
Rock 1 (Gangue)	2460	3260	0.24	1.19	30	3.5
Rock 2 (Hard gangue)	2630	12,100	0.23	3.76	42	5.1
Rock 3 (Floor)	2610	18,300	0.21	5.24	52	6.8
Rock 4 (Roof)	2600	21,500	0.19	7.17	64	7.4
Rock 5 (Inclusion)	2972	15,000	0.18	8.31	84	8.4

**Figure 2:**
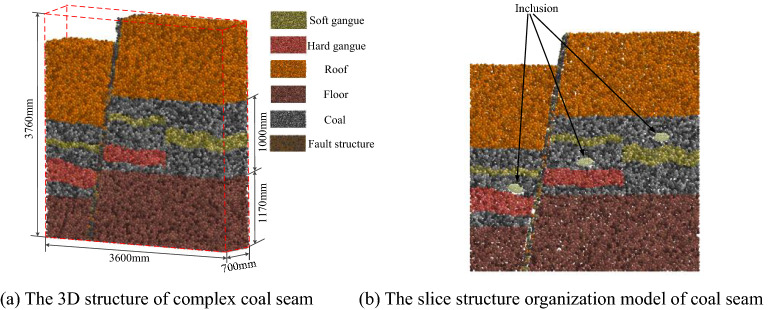
The initial basic model for high-precision 3D simulation of complex coal seam.

According to the modeling technology of irregular particle inclusions, the filling technology of coal and rock particles simulating multi mineral composition and the calculation technology of user-defined contact model of coal seam working face developed by our research group^27^, and based on the physical and mechanical property parameters of coal and rock obtained in the experimental process in Fig. 1, a high-precision 3D simulation initial basic model of 17 coal layers in Yanzhou mining area was constructed. The simulated fault structure, gangue layer, inclusion, roof and floor were randomly filled into the solid space according to the occurrence conditions. The final simulation initial basic model is shown in Fig. 2. Figure 2a shows the 3D structure of complex coal seam. Figure 2b shows the slice structure organization model of coal seam. Through the slice structure organization of its internal space, the filling shape and effect of inclusion particles in complex coal seam can be accurately displayed.

#### Update and replace of high-precision 3D simulation model structure of complex coal seam

**Figure 3:**
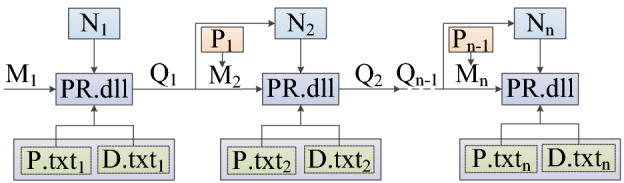
The process of structural replacement and correction.

The geological structure of complex coal seams is changeable. According to the change of coal seam data information, the 3D model needs to be reconstructed to realize data modeling and provide reliable data information for the coal and rock cutting state identification. Therefore, with the help of EDEM discrete element secondary development function, the structure of the initial basic model of high-precision 3D simulation of coal seam was update and replace to form a complex coal seam discrete element model with replaceable particles. The process of structural replacement and correction is shown in Fig. 3.

In Fig. 3, the initial basic model of coal seam can be replaced *n* times by compiling the API file of the replacement model. *N*_*n*_ is the number of times of replacement, *M*_*1*_ is the structural particle to be replaced in the initial basic model, *M*_*n*_ is the structural particle to be replaced for the nth time, *Q*_*n-1*_ is the structural particle after the nth-1st replacement, and *P*_*n*-1_ is the attribute file of the particle to be replaced for the nth time. P.txt_n_, D.txt_n_ and PR.dll are EDEM external files that need to be loaded respectively in the process of structure replacement and correction. Among the three EDEM external files, P.txt_n_ and D.txt_n_ respectively record the name, quantity, coordinate position and simulation node time before and after the replacement of structural particles, which are the particle factory information files of the replacement structure; PR.dll file is an extended library for particle replacement function in EDEM/API. It is in the form of dynamic. It can call the particle factory file information according to the instructions given at the simulation time point to complete the replacement and correction between structures.

**Figure 4:**
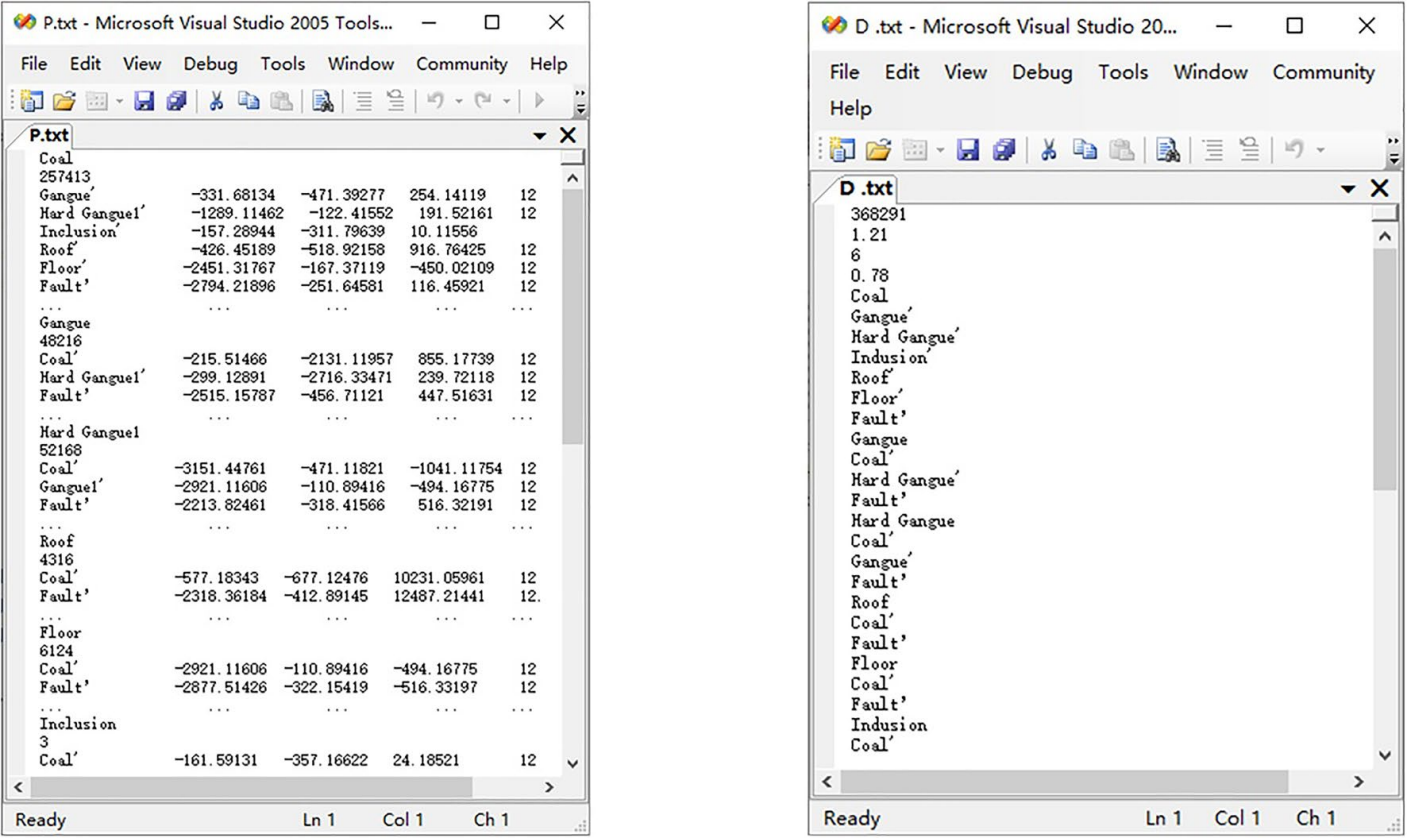
Particle factory information file of coal seam initial basic model replacement structure.

The high-precision 3D simulation model of complex coal seam needs to realize the synchronization of multiple structural particles in the process of replacement and correction. Therefore, it is necessary to control the generation of particle clusters based on the particle attribute information in the initial basic model of high-precision 3D simulation of coal seam. Based on this, the replacement model of multi types particle cluster was constructed. Firstly, the attribute information of all particle types in the initial basic model of coal seam was called respectively, so as to provide data for the compilation of multi types particle cluster replacement file. For the API of multi types particle cluster replacement, P.txt_n_ and D.txt_n_ external files need to be prepared, and their contents are shown in Fig. 4.

**Figure 5:**
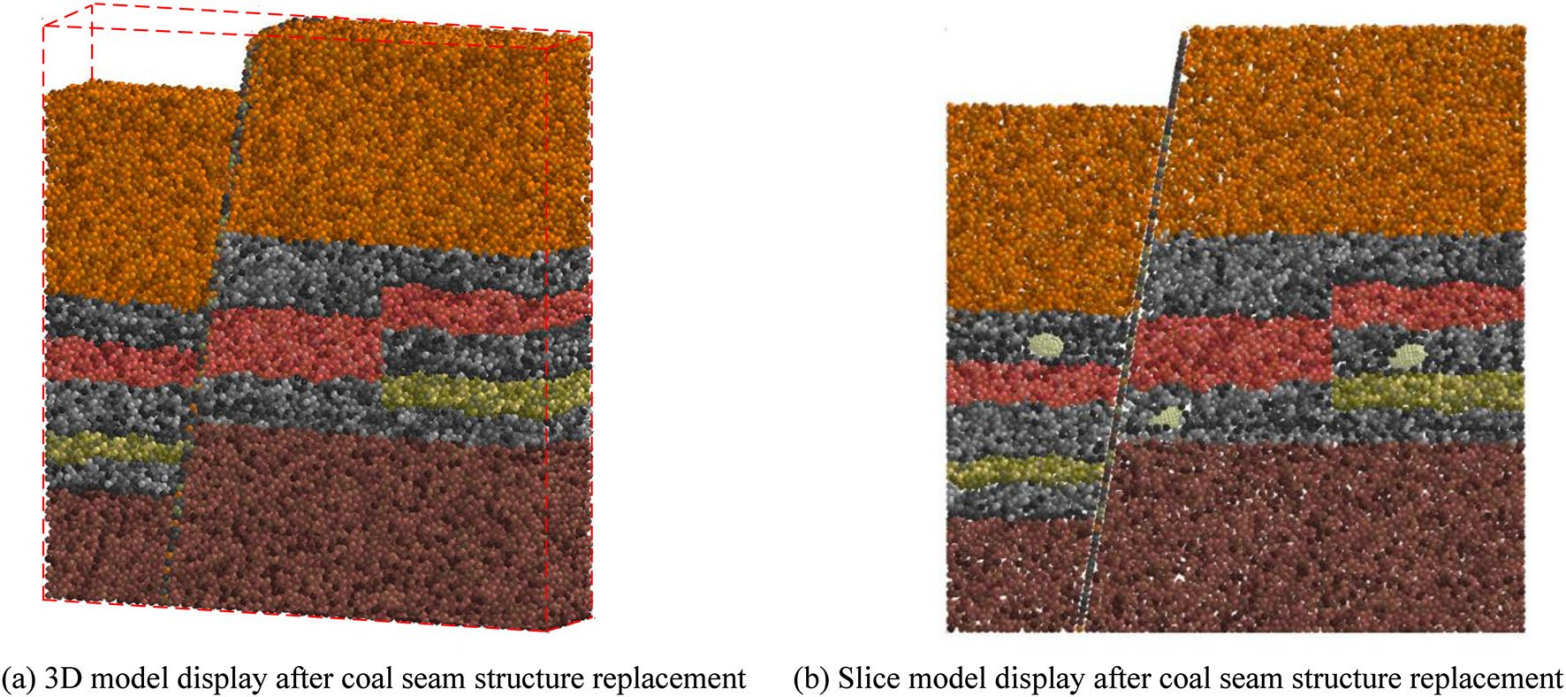
Structure replacement of initial basic model for high precision 3D simulation of coal seam.

As can be seen from Fig. 4, the P.txt_n_ and D.txt_n_ files contain the particle factory information of six types of replaced structures. Due to the huge amount of data and limited by article length, only one round of data was displayed. Using the above compiled multi types particle cluster replacement API, completed the replacement of each structure of the initial basic model of high-precision 3D simulation of coal seam, as shown in Fig. 5.

### Construction of rigid-flexible coupling virtual prototype model of shearer cutting part

A large amount of data and information support is the key problem in the construction of the coal and rock cutting state identification system. If the relevant coal and rock cutting experiments are carried out in the actual underground, the signal acquisition is difficult and has great risks. It is not only expensive but also difficult to ensure the high efficiency and high reliability of the design if the laboratory experiment is used to prepare the coal and rock wall with a large variety of working conditions. Based on this, it is an effective way to build the original database of coal and rock cutting state to simulate the cutting process of shearer using the virtual prototype model with multi-domain modeling and collaborative simulation technology as the core. Virtual prototype technology pursues a success of physical prototype. Using virtual prototype instead of physical prototype to combine it with a variety of complex intelligent algorithm strategies can solve many technical problems in the coal and rock cutting state identification.

**Figure 6:**
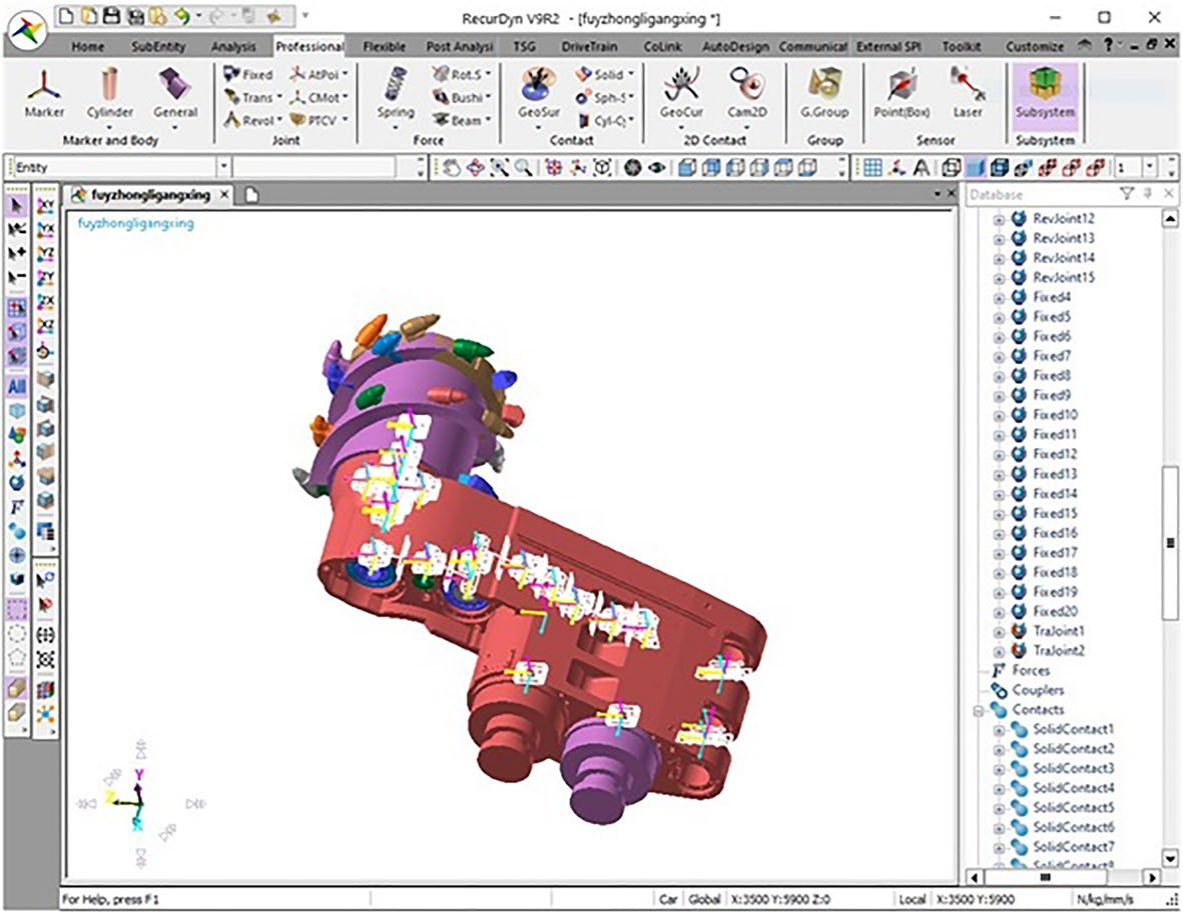
The rigid model of the shearer cutting part.

Based on the structure and material parameters of the gear transmission system of the shearer cutting part, the values of the contact parameters were added to the model. Finally the rigid model of the shearer cutting part is shown in Fig. 6.

**Figure 7:**
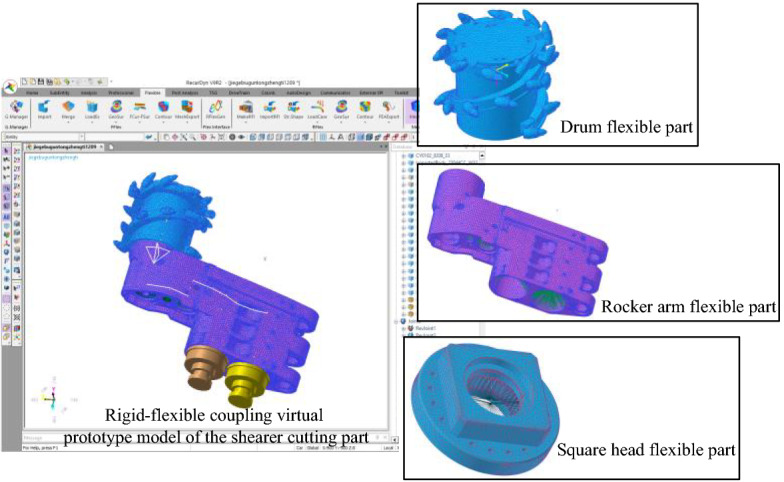
Display the rigid-flexible coupling model of shearer cutting part and flexible part of key components.

The shearer working in complex coal seam is affected by the changeable physical and mechanical properties of the cut coal and rock, and the load in its working process is nonlinear and time-varying. The rigid-flexible coupling Virtual Prototype Simulation after flexibility of key parts can improve the accuracy of vibration related information of the cutting part system. Because the flexibility of parts will greatly reduce the speed of simulation, in order to obtain accurate vibration information and improve the feasibility of simulation implementation, select the parts with obvious vibration in the cutting process of the shearer cutting part to implement flexibility. In the process of cutting and crushing, the spiral drum is in direct contact with the coal wall, and the high impact and nonlinear load lead to large vibration in the working process of the drum; The rocker arm shell is not only impacted by the meshing process of its internal gear transmission system, but also subjected to the alternating impact load generated from the working process of the spiral drum. Therefore, the rocker arm shell is the key part of vibration produced in the cutting process of the cutting part; the square head is the key part connecting the spiral drum and the output shaft. The transient impact loads produced by the spiral drum and the alternating torque produced by the output shaft will cause large vibration in the working process of the square head. Therefore, according to the above analysis, the spiral drum, rocker arm shell and square head were flexible treatment. Finally, the rigid drum, rocker arm shell and square head were replaced with flexible parts to form a rigid-flexible coupling virtual prototype model of the shearer cutting part, as shown in Fig. 7.

### Construction of the two-way coupling model for shearer cutting section to cutting complex coal seam with gangue

**Figure 8:**
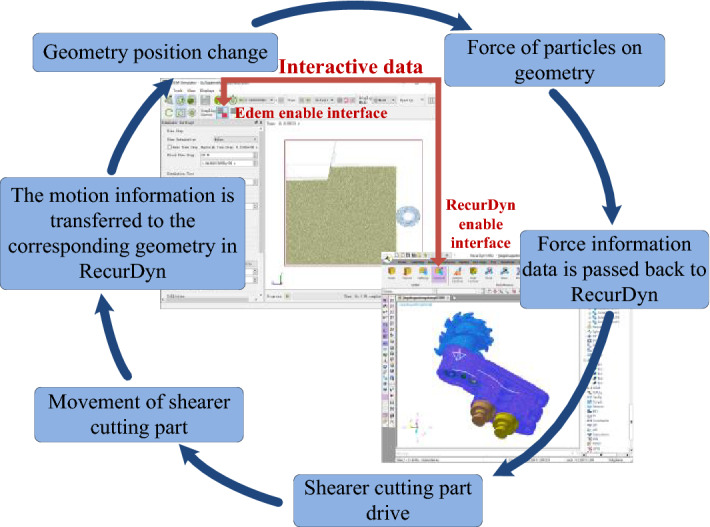
Interactive process of the two-way coupling.

The bidirectional coupling model of the cutting process of the shearer cutting part was established through the coupling interface between Edem and RecurDyn, which can realize the correlation between the high-precision three-dimensional simulation model of the complex coal seam with updated and replaced particles and the rigid-flexible coupling virtual prototype model of the cutting part. The bidirectional coupling data exchange process is shown in Fig. 8. Through DEM-MFBD interactive interface, the position information of shearer cutting part relative to coal wall working face in EDEM is transmitted to the corresponding geometry in RecurDyn, so as to realize the real-time transmission of motion information and the coal-rock state characteristic signal data. It can ensure the accuracy of simulation results.

Before the simulation of the bidirectional coupling system, the sampling frequency is determined to be 2000 Hz according to the sampling frequency theorem^28^, so the simulation step size is 0.0005 s. Set the shearer traction speed of 4 m/min, drum rotating speed of 95 r/min and the cutting depth of 630 mm to complete the bidirectional coupling simulation of the cutting process of the shearer cutting part. Using the high-precision 3D simulation model of complex coal seam and based on the replacement function of multi types particle cluster, updated the simulation model of complex coal seam and iterated repeatedly to construct 66 groups of different simulation conditions as shown in Table 2.

**Table 2:** Simulation working condition.

Firmness coefficient of coal/*f*_coal_	Firmness coefficient of rock/*f*_rock_	Coal-rock volume ratio
1.4	3.5 (Soft gangue)	0:1 1:1 1:3 3:1 1:0
	5.1 (Hard gangue)	0:1 1:1 1:3 3:1 1:0
	6.8 (Floor)	0:1 1:1 1:3 3:1 1:0
	7.4 (Roof)	0:1 1:1 1:3 3:1 1:0
	8.4 (Inclusion)	Come across inclusion
	3.5 (Soft gangue), 5.1 (Hard gangue), 6.8 (Floor), 7.4 (Roof) randomly mixed	Cross fault
2.38	3.5 (Soft gangue)	0:1 1:1 1:3 3:1 1:0
	5.1 (Hard gangue)	0:1 1:1 1:3 3:1 1:0
	6.8 (Floor)	0:1 1:1 1:3 3:1 1:0
	7.4 (Roof)	0:1 1:1 1:3 3:1 1:0
	8.4 (Inclusion)	Come across inclusion
	3.5 (Soft gangue), 5.1 (Hard gangue), 6.8 (Floor), 7.4 (Roof) randomly mixed	Cross fault
3.8	3.5 (Soft gangue)	0:1 1:1 1:3 3:1 1:0
	5.1 (Hard gangue)	0:1 1:1 1:3 3:1 1:0
	6.8 (Floor)	0:1 1:1 1:3 3:1 1:0
	7.4 (Roof)	0:1 1:1 1:3 3:1 1:0
	8.4 (Inclusion)	Come across inclusion
	3.5 (Soft gangue), 5.1 (Hard gangue), 6.8 (Floor), 7.4 (Roof) randomly mixed	Cross fault

### Data processing

**Figure 9:**
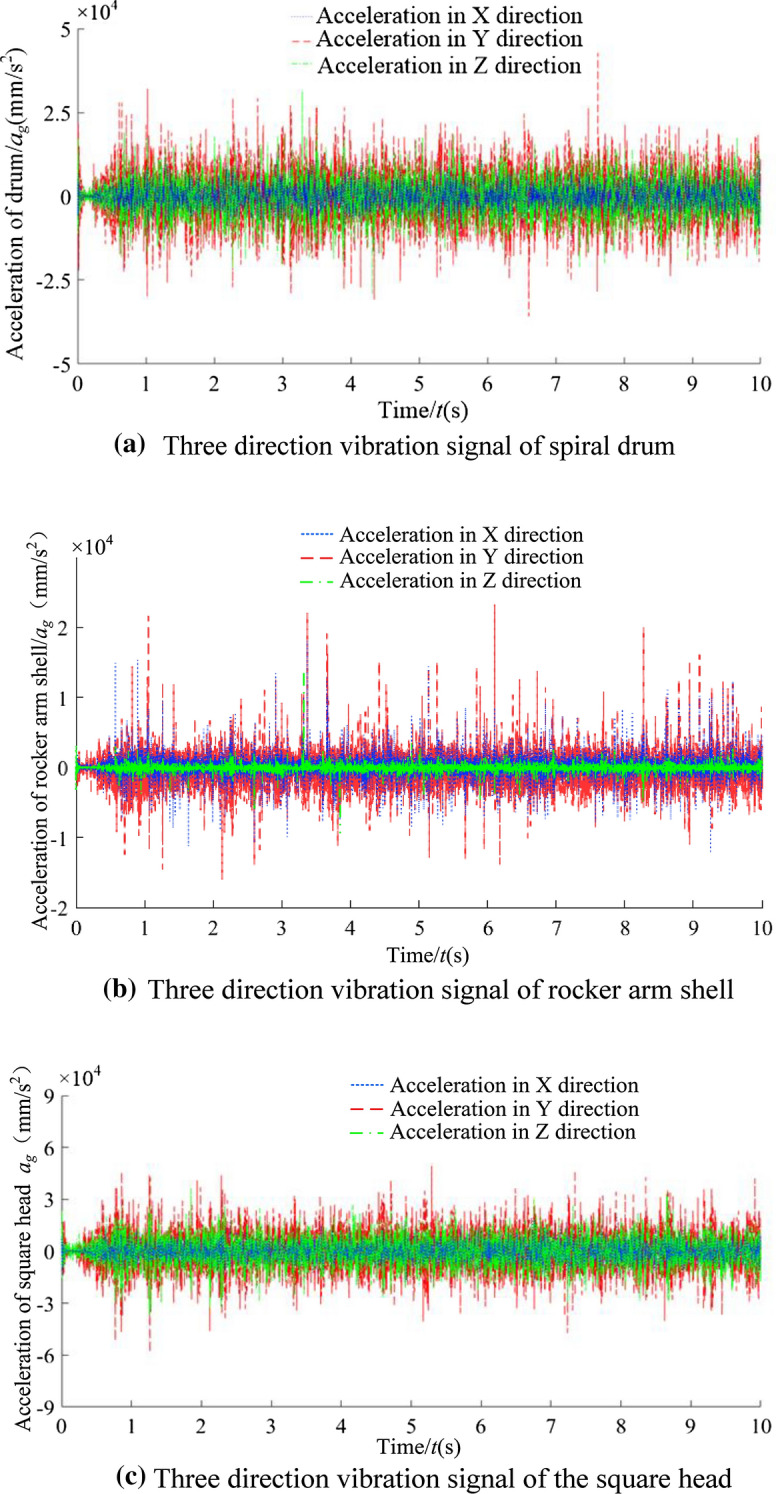
Three direction vibration signal.

The simulation data of spiral drum, rocker arm shell and square head in 66 groups of the coal and rock cutting models were extracted through the RecurDyn post-processing module. Taking one group of simulation working conditions (coal: rock = 1:3, *f*_coal_ = 2.38, *f*_rock_ = 6.8) as an example, the statistical results of vibration acceleration signals of spiral drum, rocker arm shell and square head in X, Y and Z directions are shown in Fig. 9 and Table 3.

**Table 3:** Statistical value of vibration acceleration.

Vibration acceleration(mm/s^2^)		X direction	Y direction	Z direction
Peak value	Spiral drum	6217.883	9962.288	8779.138
	Rocker arm shell	5531.017	8692.579	4896.529
	Square head	6701.245	10,889.506	8951.951
Valid values	Spiral drum	4397.371	7045.288	6208.726
	Rocker arm shell	3021.819	6338.542	2262.304
	Square head	4811.814	8235.927	6509.101
Maximum value	Spiral drum	11,310.057	42,751.179	32,405.143
	Rocker arm shell	19,037.561	21,347.322	16,851.397
	Square head	14,957.632	49,369.216	34,521.635
Minimum value	Spiral drum	− 10,162.186	− 35,853.527	− 30,084.474
	Rocker arm shell	− 11,926.454	− 18,936.411	− 9692.112
	Square head	− 11,264.238	− 58,214.506	− 33,699.728

It can be seen from Fig. 9 and Table 3 that the vibration intensity in the cutting resistance direction of the spiral drum is the largest, and the rocker arm shell and the square head are the largest in the vertical direction. In order to increase the sensitivity of the recognition system, the direction with the most severe vibration intensity of the spiral drum, rocker arm shell and square head was selected as the characteristic sample data to characterize their vibration degree.

#### Characteristic recognition and analysis of vibration signal of the spiral drum

**Figure 10:**
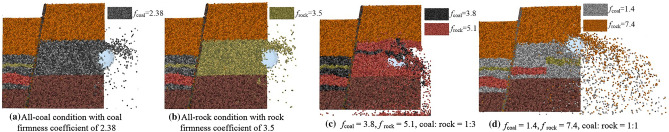
The cutting process of the shearer.

**Figure 11:**
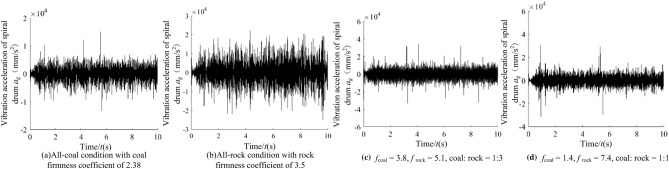
The vibration acceleration in the cutting resistance direction of the spiral drum.

Due to the limitation of article length, the 4 groups of typical working conditions were taken as examples for comparative processing and analysis. During the cutting process of the shearer (Fig. 10), the vibration acceleration in the cutting resistance direction of the spiral drum is shown in Fig. 11.

**Figure 12:**
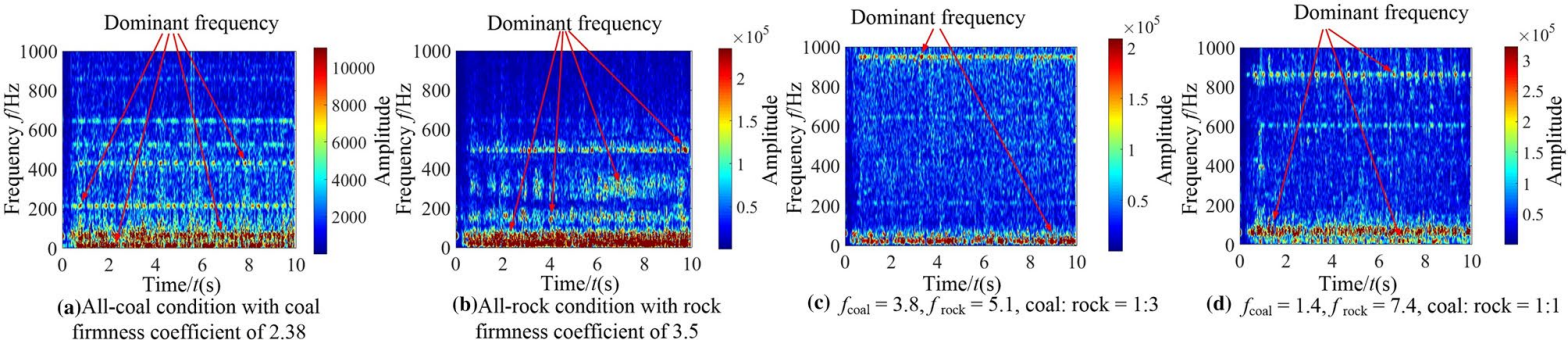
The time–frequency images of the spiral drum.

It can be seen from Fig. 11 that when there are differences in hardness and proportion of the coal and rock, the fluctuation amplitude of vibration information changes. However, their waveforms are similar and there is no significant difference. We cannot identify the specific cutting state of coal and rock only through Fig. 11. Therefore, the STFT algorithm^29,30^ defined in Eq. (1) was used to convert the vibration signal of the spiral drum, and its parameter settings are shown in Table 4. After STFT split and merge, the two-dimensional time–frequency images of the spiral drum vibration signal with the size of 128*128 under different cutting states were obtained, as shown in Fig. 12.

1 $$STFT_{x} \left( {t,f} \right) = \sum\limits_{{\delta { = }0}}^{m - 1} x \left( \delta \right)p\left( {\delta - t} \right)e^{ - j\omega \delta } d\delta$$

where, *x*(*δ*)is the original signal at time *δ*; *p*(*δ* − *t*) is the analysis window function; *m* represents the length of the window function.

**Table 4:** Parameter setting of STFT transform.

Parameter name	Parameter selection
Window type	Kaiser window
Sample length/*L*	1025
Length of window function/*L*_*t*_	256
Overlap of windows/*L*_*n*_	248

It can be seen from Fig. 12 that the difference between the coal and rock cutting states represented by STFT time–frequency image is significantly greater than that in time domain. At the same time, the time–frequency images contain richer variation features. Even if the firmness coefficient of coal is larger than that of rock, there are significant differences in the position of main frequency and the size of frequency distribution points in the time–frequency image. Under the working condition shown in Fig. 12a, the energy of the dominant frequency is mainly distributed at 10 Hz, 50 Hz, 210 Hz and 410 Hz; Under the working condition shown in Fig. 12b, the energy of the dominant frequency is distributed in the range of 0 ~ 80 Hz, 150 Hz, 320 Hz and 500 Hz respectively; Under the working condition shown in Fig. 12c, the energy of the dominant frequency is distributed in the range of 0 ~ 50 Hz and 960 Hz respectively, and its energy is 2.14 × 10^5^; Under the working condition shown in Fig. 12d, the energy of the dominant frequency is distributed at 10 Hz, 70 Hz and 880 Hz respectively, and its energy is 3.57 × 10^5^.This is mainly due to the differences in amplitude, period and other characteristics of the vibration signal obtained by the shearer spiral drum under different cutting states, and the STFT time–frequency image can highlight the feature differences. Therefore, through the change of STFT, we can make full use of the information of signal in time domain and frequency domain, and lay a foundation for the recognition performance of the coal and rock cutting state recognition system.

#### Characteristic recognition and analysis of vibration signal of the rocker arm shell

**Figure 13:**
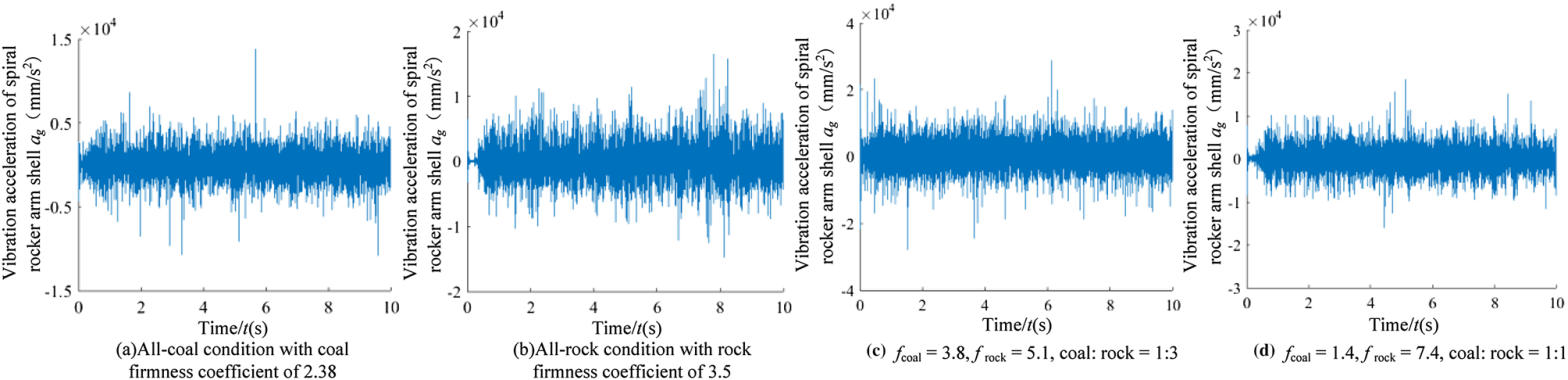
The time–frequency images of the rocker arm shell.

In order to analyze the identification characteristics of the vibration signal of the rocker arm shell, corresponding to the four typical complex working conditions in Section “Characteristic recognition and analysis of vibration signal of the spiral drum”, the vibration acceleration of the rocker arm shell in the vertical direction during the cutting process of the shearer was extracted, as shown in Fig. 13.

**Figure 14:**
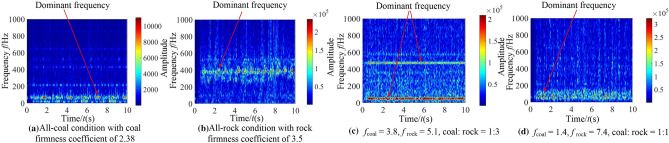
The time–frequency images of the rocker arm shell.

According to Fig. 13, the information of the coal and rock cutting state under different working conditions will show unstable changes in varying degrees in the vibration acceleration of rocker arm shell. This difference becomes the basis for using the vibration signal of the rocker arm shell to characterize the cutting state of different coal and rock. However, the singleness of time domain analysis will lead to poor description of the coal and rock cutting state characteristics and reduce the accuracy of identification. Therefore, the STFT transform was also used to convert the time-domain signal of the rocker arm housing into a spectrum image to synthesize the energy characteristics of time–frequency domain and make up for the defects of time domain information, as shown in Fig. 14.

The time–frequency resolution of the vibration information of the rocker arm shell is well retained by STFT transformation. At the same time, the characteristic information of energy clusters is prominent. The variation and distribution of energy characteristics under different working conditions are obviously different. Therefore, in the process of building the recognition system, the time–frequency image can significantly improve the effectiveness of using the vibration signal samples of the rocker arm shell.

#### Characteristic recognition and analysis of vibration signal of the square head

**Figure 15:**
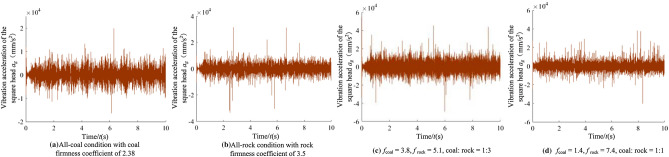
The time–frequency images of the square head.

Taking the four typical complex working conditions corresponding to Section “Characteristic recognition and analysis of vibration signal of the spiral drum” as an example, the vibration acceleration curve of the square head during shearer cutting is shown in Fig. 15.

**Figure 16:**
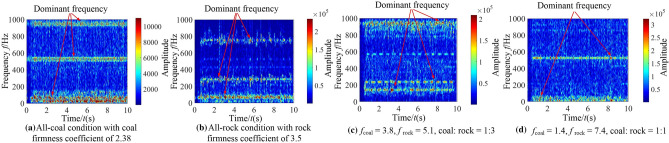
The time–frequency images of the square head.

As can be seen from Fig. 15, the vibration signal of the square head under different working conditions varies in time domain. However, due to the influence of vibration and shock, it belongs to unstable signal, and there is similarity between waveforms. The lack of prominent vibration characteristics makes the learning process of the coal and rock cutting state characteristics more difficult. Therefore, in order to enhance the effectiveness of feature information extraction, STFT transform was also used to convert the one-dimensional time domain signal of the square head into the two-dimensional time–frequency image with time and frequency resolution of 128, as shown in Fig. 16.

The dominant frequency in the time–frequency diagram under each working condition was marked in Fig. 16. It can be seen from Fig. 16 that there are obvious differences in the dominant frequency energy position, range size, characteristic group shape and other information in the square head vibration signal between different working conditions. Due to the participation of STFT transform, it plays the role of fully extracting energy features, retains the energy features near the actual frequency of the signal under various working conditions, and obtains the time–frequency information with strong focusing. The image not only has good frequency resolution, but also has high discrimination of each frequency component point. This shows that the square head vibration signal is transformed by the method of STFT, which provides the original samples with high availability for the training of deep learning. Using the time–frequency image of the square head vibration signal to characterize the cutting state characteristics of coal and rock is very necessary to improve the accuracy of recognition system.

## Vibration feature fusion of the coal and rock cutting based on MW

### Image fusion

**Figure 17:**
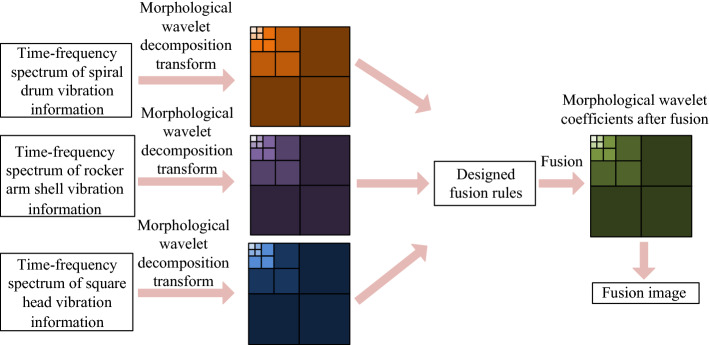
Fusion framework of vibration characteristics of the coal and rock cutting based on MW.

The time–frequency spectrum images of spiral drum, rocker arm shell and square head under different coal and rock cutting conditions contain a lot of details. In order to make the time–frequency spectrum images under various working conditions more representative and strengthen the discrimination of the original samples in the database, the nonlinear characteristics of morphological technology and the multi-level characteristics of wavelet transform decomposition were combined to implement the image fusion of morphological wavelet transform (MW) for the time–frequency spectrum images of the Vibration information of the three under the same working conditions. The specific framework of vibration information fusion is shown in Fig. 17.

Let the time–frequency images of vibration signals of the spiral drum, the rocker arm shell and the square head under different the coal and rock cutting conditions be *M*_1_^*x*^, *M*_2_^*x*^ and *M*_3_^*x*^ respectively (where *x* is the serial number of the coal and rock cutting working conditions, *x* = 1,2,…,24), and decompose the image (where *i* = 1,2,3) *n* times:

2 $$\begin{gathered} \lambda_{\varpi }^{ \uparrow } (M^{\varpi } ) = M^{\varpi + 1} ,M^{\varpi + 1} \in X_{\varpi + 1} \hfill \\ \vartheta_{\varpi }^{ \uparrow } (M^{\varpi } ) = U^{\varpi + 1} ,U^{\varpi + 1} \in Y_{\varpi + 1} \hfill \\ M_{i}^{x} = \left. {\left\{ {M_{i}^{n} ,U_{i}^{1} ,U{}_{i}^{2} , \cdots U_{i}^{n} } \right.} \right\} \hfill \\ \end{gathered}$$

where,* M*^*ϖ*^ ∈ *X*_*ϖ*_ ; X_*ϖ*_ → X_*ϖ*+1_ is the analysis signal decomposition space of the information in the image; X_*ϖ*_ → Y_*ϖ*+1_ is the analysis detail decomposition space of the information in the image; λ_ϖ_^↑^ is the analysis signal operator of the information decomposition in the image; ϑ_ϖ_^↑^ is the analysis detail operator of the information decomposition in the image; *M*_*i*_^*n*^ is the analysis signal coefficient after image; *M*_*i*_^*x*^ is decomposed *n* times; *U*_*i*_^*n*^ is the analysis detail coefficient of the image after *M*_*i*_^*x*^ decomposition *n* times.

Fusion rule of image *M*_*i*_^*x*^ at low frequency:

3 $$\begin{gathered} \alpha_{1}^{n} = \frac{{M_{1}^{xn} \left( {p,q} \right)}}{{M_{1}^{xn} \left( {p,q} \right) + M_{2}^{xn} \left( {p,q} \right) + M_{3}^{xn} \left( {p,q} \right)}} \hfill \\ \alpha_{2}^{n} \left( {p,q} \right) = \frac{{M_{2}^{xn} \left( {p,q} \right)}}{{M_{1}^{xn} \left( {p,q} \right) + M_{2}^{xn} \left( {p,q} \right) + M_{3}^{xn} \left( {p,q} \right)}} \hfill \\ \alpha_{3}^{n} \left( {p,q} \right) = \frac{{M_{3}^{xn} \left( {p,q} \right)}}{{M_{1}^{xn} \left( {p,q} \right) + M_{2}^{xn} \left( {p,q} \right) + M_{3}^{xn} \left( {p,q} \right)}} \hfill \\ \end{gathered}$$

4 $$\begin{gathered} M^{n} \,\left( {p,q} \right) = \alpha_{1}^{n} \left( {p,q} \right) \cdot M_{1}^{xn} \left( {p,q} \right) + \alpha_{2}^{n} \times \left( {p,q} \right) \cdot M_{2}^{xn} \left( {p,q} \right) + \alpha_{3}^{n} \left( {p,q} \right) \cdot M_{3}^{xn} \left( {p,q} \right) \hfill \\ \cdot \hfill \\ \end{gathered}$$

where, *α*_*i*_^*n*^(*p*,*q*)(*i* = 1,2,3) is the weighted coefficient of the image; *M*^*n*^(*p*,*q*) is the low frequency coefficient of the fused image; *M*(*p*,*q*) is the position of image structure element.

The high-frequency components of image *M*_*i*_^*x*^ in the horizontal, vertical and diagonal directions follow the pyramid contrast fusion rule. If the decomposition scale *Y* is within the range of the highest decomposition scale *T*, then:

5 $$\left\{ \begin{gathered} e_{a}^{Y} (M_{i}^{x} )(m,n) = e_{a}^{Y} (M_{1}^{x} )(m,n)\begin{array}{*{20}c} {} & {} \\ \end{array} if\begin{array}{*{20}c} {} \\ \end{array} B_{a}^{Y} (M_{1}^{x} )(m,n) \ge B_{a}^{Y} (M_{2}^{x} )(m,n),B_{a}^{Y} (M_{3}^{x} )(m,n) \hfill \\ e_{a}^{Y} (M_{i}^{x} )(m,n) = e_{a}^{Y} (M_{2}^{x} )(m,n)\begin{array}{*{20}c} {} & {} \\ \end{array} if\begin{array}{*{20}c} {} \\ \end{array} B_{a}^{Y} (M_{2}^{x} )(m,n) \ge B_{a}^{Y} (M_{1}^{x} )(m,n),B_{a}^{Y} (M_{3}^{x} )(m,n) \hfill \\ e_{a}^{Y} (M_{i}^{x} )(m,n) = e_{a}^{Y} (M_{3}^{x} )(m,n)\begin{array}{*{20}c} {} & {} \\ \end{array} if\begin{array}{*{20}c} {} \\ \end{array} B_{a}^{Y} (M_{3}^{x} )(m,n) \ge B_{a}^{Y} (M_{1}^{x} )(m,n),B_{a}^{Y} (M_{2}^{x} )(m,n) \hfill \\ \end{gathered} \right.$$

6 $$\left\{ \begin{gathered} e_{b}^{Y} (M_{i}^{x} )(m,n) = e_{b}^{Y} (M_{1}^{x} )(m,n)\begin{array}{*{20}c} {} & {} \\ \end{array} if\begin{array}{*{20}c} {} \\ \end{array} B_{b}^{Y} (M_{1}^{x} )(m,n) \ge B_{b}^{Y} (M_{2}^{x} )(m,n),B_{b}^{Y} (M_{3}^{x} )(m,n) \hfill \\ e_{b}^{Y} (M_{i}^{x} )(m,n) = e_{b}^{Y} (M_{2}^{x} )(m,n)\begin{array}{*{20}c} {} & {} \\ \end{array} if\begin{array}{*{20}c} {} \\ \end{array} B_{b}^{Y} (M_{2}^{x} )(m,n) \ge B_{b}^{Y} (M_{1}^{x} )(m,n),B_{b}^{Y} (M_{3}^{x} )(m,n) \hfill \\ e_{b}^{Y} (M_{i}^{x} )(m,n) = e_{b}^{Y} (M_{3}^{x} )(m,n)\begin{array}{*{20}c} {} & {} \\ \end{array} if\begin{array}{*{20}c} {} \\ \end{array} B_{b}^{Y} (M_{3}^{x} )(m,n) \ge B_{b}^{Y} (M_{1}^{x} )(m,n),B_{b}^{Y} (M_{2}^{x} )(m,n) \hfill \\ \end{gathered} \right.$$

7 $$\left\{ \begin{gathered} e_{c}^{Y} (M_{i}^{x} )(m,n) = e_{c}^{Y} (M_{1}^{x} )(m,n)\begin{array}{*{20}c} {} & {} \\ \end{array} if\begin{array}{*{20}c} {} \\ \end{array} B_{c}^{Y} (M_{1}^{x} )(m,n) \ge B_{c}^{Y} (M_{2}^{x} )(m,n),B_{c}^{Y} (M_{3}^{x} )(m,n) \hfill \\ e_{c}^{Y} (M_{i}^{x} )(m,n) = e_{c}^{Y} (M_{2}^{x} )(m,n)\begin{array}{*{20}c} {} & {} \\ \end{array} if\begin{array}{*{20}c} {} \\ \end{array} B_{c}^{Y} (M_{2}^{x} )(m,n) \ge B_{c}^{Y} (M_{1}^{x} )(m,n),B_{c}^{Y} (M_{3}^{x} )(m,n) \hfill \\ e_{c}^{Y} (M_{i}^{x} )(m,n) = e_{c}^{Y} (M_{3}^{x} )(m,n)\begin{array}{*{20}c} {} & {} \\ \end{array} if\begin{array}{*{20}c} {} \\ \end{array} B_{c}^{Y} (M_{3}^{x} )(m,n) \ge B_{c}^{Y} (M_{1}^{x} )(m,n),B_{c}^{Y} (M_{2}^{x} )(m,n) \hfill \\ \end{gathered} \right.$$

where, *a* is the horizontal direction; *b* is the vertical direction; *c* is the diagonal direction; *B*_*a*_^*Y*^ is the contrast ratio of the image in the horizontal direction; *B*_*b*_^*Y*^ is the contrast ratio of the image in the vertical direction; *B*_*c*_^*Y*^ is the contrast ratio of the image in the diagonal direction.

If *Y* ∉ *T*, then:

8 $$\left\{ \begin{gathered} e_{a}^{Y} (M_{i}^{x} )(m,n) = e_{a}^{Y} (M_{1}^{x} )(m,n)\begin{array}{*{20}c} {} \\ \end{array} if\begin{array}{*{20}c} {} \\ \end{array} Std_{a}^{Y} (M_{1}^{x} )(m,n) \ge Std_{a}^{Y} (M_{2}^{x} )(m,n),Std_{a}^{Y} (M_{3}^{x} )(m,n) \hfill \\ e_{a}^{Y} (M_{i}^{x} )(m,n) = e_{a}^{Y} (M_{2}^{x} )(m,n)\begin{array}{*{20}c} {} \\ \end{array} if\begin{array}{*{20}c} {} \\ \end{array} Std_{a}^{Y} (M_{2}^{x} )(m,n) \ge Std_{a}^{Y} (M_{1}^{x} )(m,n),Std_{a}^{Y} (M_{3}^{x} )(m,n) \hfill \\ e_{a}^{Y} (M_{i}^{x} )(m,n) = e_{a}^{Y} (M_{3}^{x} )(m,n)\begin{array}{*{20}c} {} \\ \end{array} if\begin{array}{*{20}c} {} \\ \end{array} Std_{a}^{Y} (M_{3}^{x} )(m,n) \ge Std_{a}^{Y} (M_{1}^{x} )(m,n),Std_{a}^{Y} (M_{2}^{x} )(m,n) \hfill \\ \end{gathered} \right.$$

9 $$\left\{ \begin{gathered} e_{b}^{Y} (M_{i}^{x} )(m,n) = e_{b}^{Y} (M_{1}^{x} )(m,n)\begin{array}{*{20}c} {} \\ \end{array} if\begin{array}{*{20}c} {} \\ \end{array} Std_{b}^{Y} (M_{1}^{x} )(m,n) \ge Std_{b}^{Y} (M_{2}^{x} )(m,n),Std_{b}^{Y} (M_{3}^{x} )(m,n) \hfill \\ e_{b}^{Y} (M_{i}^{x} )(m,n) = e_{b}^{Y} (M_{2}^{x} )(m,n)\begin{array}{*{20}c} {} \\ \end{array} if\begin{array}{*{20}c} {} \\ \end{array} Std_{b}^{Y} (M_{2}^{x} )(m,n) \ge Std_{b}^{Y} (M_{1}^{x} )(m,n),Std_{b}^{Y} (M_{3}^{x} )(m,n) \hfill \\ e_{b}^{Y} (M_{i}^{x} )(m,n) = e_{b}^{Y} (M_{3}^{x} )(m,n)\begin{array}{*{20}c} {} \\ \end{array} if\begin{array}{*{20}c} {} \\ \end{array} Std_{b}^{Y} (M_{3}^{x} )(m,n) \ge Std_{b}^{Y} (M_{1}^{x} )(m,n),Std_{b}^{Y} (M_{2}^{x} )(m,n) \hfill \\ \end{gathered} \right.$$

10 $$\left\{ \begin{gathered} e_{c}^{Y} (M_{i}^{x} )(m,n) = e_{c}^{Y} (M_{1}^{x} )(m,n)\begin{array}{*{20}c} {} \\ \end{array} if\begin{array}{*{20}c} {} \\ \end{array} Std_{c}^{Y} (M_{1}^{x} )(m,n) \ge Std_{c}^{Y} (M_{2}^{x} )(m,n),Std_{c}^{Y} (M_{3}^{x} )(m,n) \hfill \\ e_{c}^{Y} (M_{i}^{x} )(m,n) = e_{c}^{Y} (M_{2}^{x} )(m,n)\begin{array}{*{20}c} {} \\ \end{array} if\begin{array}{*{20}c} {} \\ \end{array} Std_{c}^{Y} (M_{2}^{x} )(m,n) \ge Std_{c}^{Y} (M_{1}^{x} )(m,n),Std_{c}^{Y} (M_{3}^{x} )(m,n) \hfill \\ e_{c}^{Y} (M_{i}^{x} )(m,n) = e_{c}^{Y} (M_{3}^{x} )(m,n)\begin{array}{*{20}c} {} \\ \end{array} if\begin{array}{*{20}c} {} \\ \end{array} Std_{c}^{Y} (M_{3}^{x} )(m,n) \ge Std_{c}^{Y} (M_{1}^{x} )(m,n),Std_{c}^{Y} (M_{2}^{x} )(m,n) \hfill \\ \end{gathered} \right.$$

where, *Std* is the standard deviation of the central area of the image pixel.

Finally, the time–frequency image of the vibration signal representing the cutting state of coal and rock was synthesized by using the fusion rules, which is expressed as:

11 $$M^{\varpi } (V,W) = \lambda_{\varpi }^{ \downarrow } \left[ {M^{\varpi + 1} (V,W)} \right] + \vartheta_{\varpi }^{ \downarrow } \left[ {U^{\varpi + 1} (V,W)} \right]$$

where, (*V*,*W*) ∈ (*p*,*q*); *λ*_*ϖ*_^↓^ is the analysis signal operator of the information synthesis in the image; *ϑ*_ϖ_^↓^ is the analysis detail operator of the information synthesis in the image.

**Figure 18:**
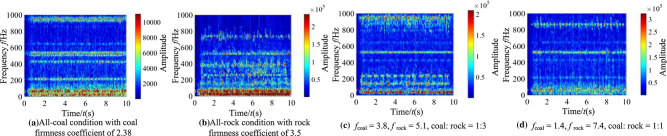
Time-spectrum fusion image of vibration information under different working conditions.

Based on MW image fusion model, the time–frequency spectrum images of spiral drum, rocker arm shell and square head under different coal and rock cutting conditions were fused and transformed. Due to the variety of working conditions set, the original image samples of the database are large. Therefore, Fig. 18 only shows fusion image samples corresponding to the 4 typical working conditions in Section “Construction of high-precision 3D simulation model for complex coal seam”. It can be seen from Fig. 18 that the MW time–frequency spectrum image is used for fusion, which better retains the feature information in the original image, and realizes the fusion of vibration information between different parts of the shearer cutting part that represents the same coal and rock cutting state. This will not only increase the amount of information of the original basic samples in the database, but also help to improve the recognition ability of the coal and rock cutting status.

### Comparative analysis of experiments

**Figure 19:**
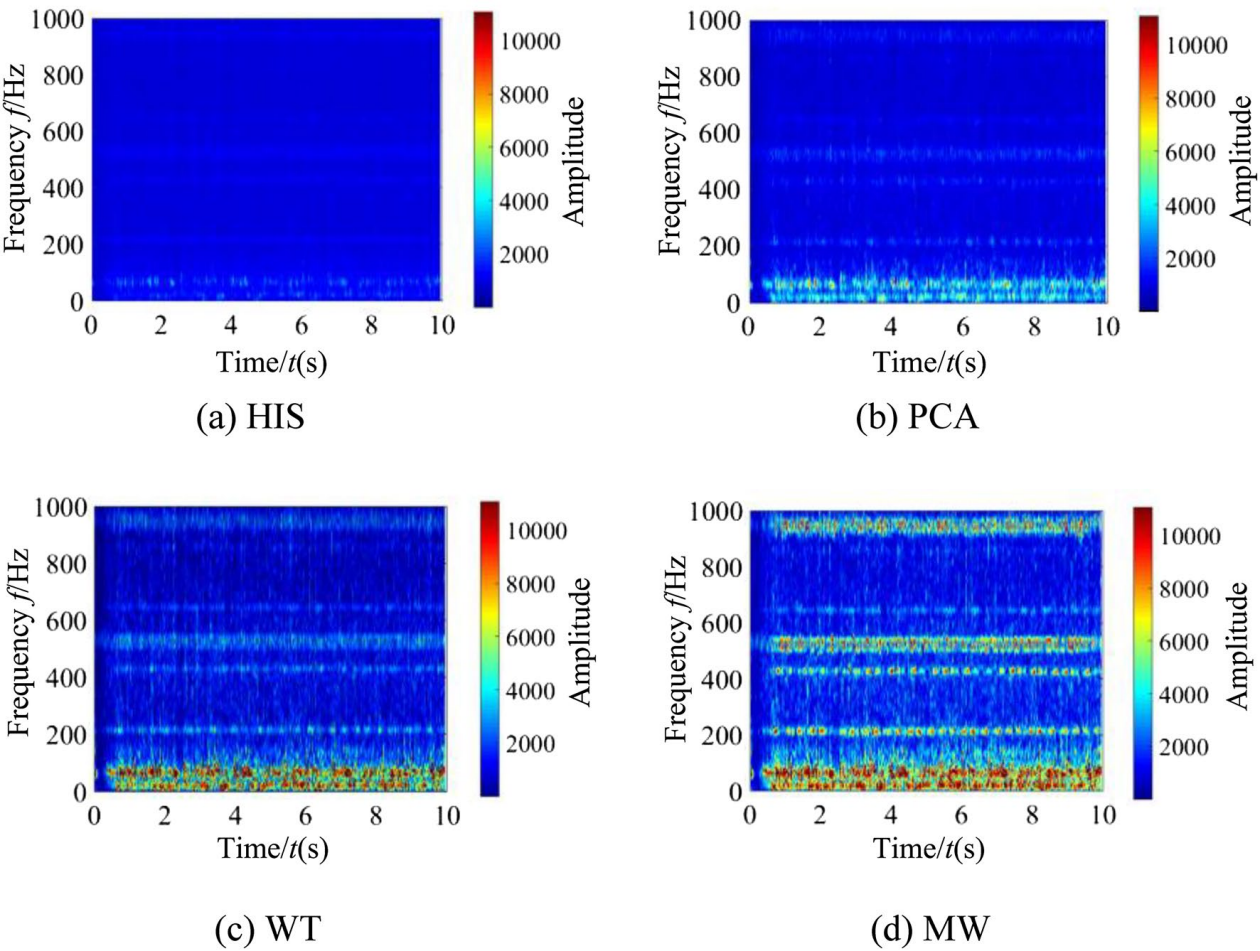
Fused images under different methods.

In order to verify the superiority of the fusion model of coal and rock cutting vibration characteristics, a group of all coal cutting working conditions with the firmness coefficient of 2.38 were selected at random. The time–frequency spectrum images of the vibration information of the spiral drum, rocker arm shell and square head during the shearer cutting process were extracted, and the image features were fused using MW, HIS (Hue Intensity Saturation), PCA (Principal Component Analysis) and WT (Wavelet Transform) models respectively. In the process of fusion, four layers of decomposition were adopted, and finally feature fusion images with the size of 128 * 128 were obtained in different methods, as shown in Fig. 19.

It can be seen from the fusion image effect of Fig. 19 that the fusion effect of WT model is better than that of HIS and PCA models. The image after WT model fusion has improved its smoothness and clarity. However, due to the different sensitivity of WT model to bright and dim spots, the brightness effect of the image is relatively poor. At the same time, the high-frequency features in the time–frequency spectrum images of the spiral drum, rocker arm shell and square head are obviously lost. The image fused by MW model has moderate brightness and high fit with the original image. The image is clearer than the other three methods. At the same time, the dominant frequency features in the time–frequency spectrum images of the spiral drum, rocker arm shell and square head are highly fused and retained. The image degradation is improved and the feature fusion effect is better.

In order to further carry out quantitative evaluation on the fusion model of coal and rock cutting vibration characteristics based on MW, the evaluation indicators of the four models were counted, and the comparison results are shown in Table 5.

**Table 5:** Comparative analysis of evaluation indexes of four fusion models.

Evaluation indexes	HIS	PCA	WT	MW
Standard deviation	0.1521	0.1577	0.1942	0.2011
Articulation	3.8127	4.7622	5.3161	5.9785
Signal to noise ratio	1.2144	1.5136	5.2273	7.1484
Average error	24.1194	23.2712	15.3353	12.6026
Mutual information	1.1146	1.1383	1.1757	1.1902

According to the statistical results in Table 5, the SD value of the image fused by MW model is the largest. This shows that the discrete distribution degree of gray level of time–frequency spectrum image representing the coal and rock cutting state after MW change fusion is higher than that of other three fusion models. The fused image has the largest contrast and the richest feature information. The AG value of MW model is greater than that of other three fusion models. This shows that using MW fusion method, the subtle features in the source image achieve fusion transformation, which can increase the representativeness of the coal and rock cutting state. Compared with HIS and PCA models, the SNR value of WT model fusion image is significantly improved, but its effect is not good compared with MW model, and the fusion effect of MW model is the best. It can be seen from the comparison of AE value that MW transform improves the ability of model fusion image information feature points, and the effectiveness of the fused image to represent the coal and rock cutting state is obviously stronger than HIS, PCA and WT models. It can be seen from the MI values of each model that the MI value of MW model is the largest. This shows that the fused image has a higher coincidence degree with the time–frequency spectrum original image of the spiral drum, rocker arm shell and square head, and the ability to retain the feature information in the source image is the strongest. Based on the fusion ability evaluation indicators of the four models, the image fusion comprehensive performance of MW model is the best, which objectively verified the effectiveness of the MW based the coal and rock cutting vibration feature fusion model.

## Extension of the coal and rock cutting state samples by improved DCGAN network

### Design of improved DCGAN network

**Figure 20:**
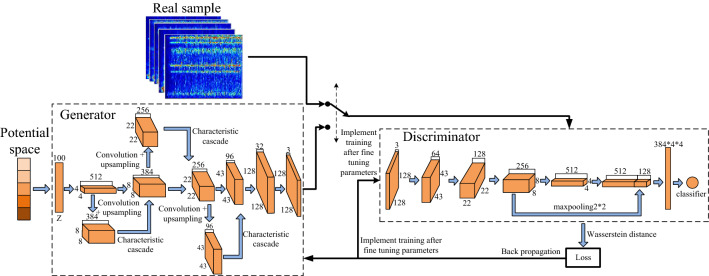
Improved DCGAN model.

During the training of coal and rock cutting state recognition network, if there are too few samples, it is easy to have overfitting phenomenon, resulting in the decline of recognition accuracy. Therefore, a large number of data samples must be obtained. The improved DCGAN model based on GAN (Generic Advantageous Networks) network was selected to generate high-quality time–frequency spectrum samples, enrich the original data set, improve the stability and robustness of the model, and ensure the quality of generated samples. The improved DCGAN model is composed of generator *G* and discriminator *D*, and its network structure is shown in Fig. 20. It can be seen from Fig. 20 that in the generator model of the improved DCGAN network, a 4-layer deep learning network with transposed convolution structure was used to connect its input layer and output layer to ensure that the characteristic graph completes the dimensional transformation while improving the stability of the system model training. Each layer of convolution structure in the generator model used convolution kernels and convolution steps of different sizes to complete the reverse convolution of its characteristic image, so as to improve the resolution of synthetic samples in the coal and rock cutting state. At the same time, in order to enrich the semantic information of the coal and rock cutting state feature map, and made the synthetic sample more close to the real sample, the convolution was performed again after the reverse convolution learning for each convolution layer, and the upper sampling was completed. During the implementation of up-sampling, bilinear interpolation was used to improve the ability of the network to effectively retain the edge information of the spectrum image in the coal and rock cutting state. In the discriminator model of the improved DCGAN network, five layers of network learning structure were set, one layer of fusion feature information structure was added to the traditional DCGAN network, and the other four layers were network learning layers with convolution structure. Like the generator, the convolution kernel and convolution step size of each layer of convolution structure of the discriminator are also different. The added fusion feature information structure layer is located behind the four-layer convolution structure. After the feature map in the third-layer convolution is maximally pooled, it is fused with the feature map in the fourth-layer convolution to uniformly normalize it into the feature map of the same size, which improves the ability of the discriminator to distinguish the true and false images, quickly carries out comparative identification, and indirectly promotes the ability of the generator to synthesize high-quality coal and rock cutting state samples. The detailed parameters of generator *G* and discriminator* D*, as shown in Table 6.

**Table 6:** Structural parameters of generator and discriminator models.

Generator	Enter channel dimension	Output channel dimension	Input dimension	Output dimension	Convolution kernel dimension	Step
deconv1	512	384	4*4	8*8	3*3	2
conv-upsample1	384	128	8*8	22*22	3*3	3
deconv2	384	128	8*8	22*22	3*3	3
concact1	Feature connection	256	Feature connection	22*22	\	\
deconv3	256	48	22*22	43*43	5*5	2
conv-upsample2	256	48	22*22	43*43	5*5	2
concact2	Feature connection	96	Feature connection	43*43	\	\
deconv4	96	16	43*43	128*128	11*11	3
conv-upsample3	96	16	43*43	128*128	11*11	3
concact3	Feature connection	32	Feature connection	128*128	\	\
fc	32	3	128*128	128*128	1*1	1

Discriminator	Enter channel dimension	Output channel dimension	Input dimension	Output dimension	Convolution kernel dimension	Step
conv1	3	64	128*128	43*43	7*7	3
conv2	64	128	43*43	22*22	5*5	2
conv3	128	256	22*22	8*8	3*3	3
conv4	256	512	8*8	4*4	3*3	2
maxpooling	256	256	8*8	4*4	2*2	2
maxpooling	256	256	8*8	4*4	2*2	2

### Training of improved DCGAN network

Before generating synthesis samples, the model needs to be trained. After training, the generator model and discriminator model in the sample expansion DCGAN network of the coal and rock cutting state need to achieve the optimal output goal, and its goal is shown in formula (12):

12 $$\begin{gathered} \mathop {\max }\limits_{D} V\left( {D,G} \right) = E_{{x\sim P_{data(x)} }} \left[ {\log \left( {D\left( x \right)} \right)} \right] + E_{{z\sim p_{z} (z)}} \left[ {\log \left( {1 - D\left( {G\left( z \right)} \right)} \right)} \right] \hfill \\ \mathop {\min }\limits_{G} V\left( {D,G} \right) = E_{{z\sim p_{z} (z)}} \left[ {\log \left( {1 - D(G(z))} \right)} \right] \hfill \\ \end{gathered}$$

where, log*D*(*x*) is the comparison and identification output of the discriminator to the real sample after the training process; log(1-*D*(*G*(*z*))) is the data synthesized by the generator after the training process.

In order to improve the robustness of the sample expansion model of the coal and rock cutting state and improve the ability of the generator to synthesize high-quality samples, a gradient penalty was added to the improved DCGAN network. The model with gradient penalty term can ensure the continuity of Lipschitz function constraints, effectively solve the problem of unbalanced weight distribution in the training process. While preserving the characteristic details of the vibration time–frequency samples in the real original, the goal of expanding the samples can be achieved. The mathematical model of gradient penalty is shown in formula (13):

13 $$GP = \gamma E_{{a^{^{\prime}} \sim P_{{a^{^{\prime}} }} }} \left[ {\left\| {\nabla a^{^{\prime}} } \right.\left. {D\left( {a^{^{\prime}} } \right)} \right\|_{p} - 1} \right]^{2}$$

where, *a’* is the sampling point distributed in the synthetic data sample; ||∇*a’ D*(*a’*)|| is the gradient expression of the discriminator.

In order to enhance the stability of the system in the training process, the improved model also changed the contrastive discrimination criterion of the discriminator on the basis of the traditional DCGAN network training process. Wasserstein distance was used as the criterion to evaluate the sample type:

14 $$W\left( {P_{data} ,P_{g} } \right) = \mathop {\inf }\limits_{{\lambda \sim \prod \left( {P_{data} ,P_{g} } \right)}} E_{{\left( {x,y} \right) \sim \lambda }} \left[ {\left\| {a - \left. b \right\|} \right.} \right]$$

where, *W*(*P*_*data*_, *P*_*g*_) is the Wasserstein distance distributed between the input real original data and the synthetic data; inf() is the infimum for solving the set data; Π(*P*_*data*_, *P*_*g*_) is the scattered set of input real original data and synthetic data; *λ* is the possible unified and decentralized between the input real original data and synthetic data; *a* is the real original data; *b* is the synthesize data.

**Figure 21:**
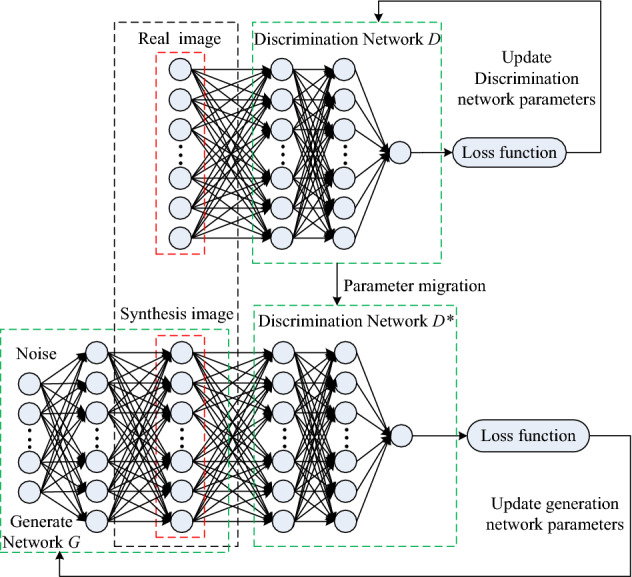
Overall flow diagram of coal and rock cutting state sample augmented DCGAN network training.

The generator model of the improved DCGAN network read the vibration time–frequency samples representing the cutting state of coal and rock. At the same time, the generator model learns and trains the data distribution law of its samples through convolution structure, and then synthesizes new feature samples. In the training process, the discriminator model makes use of the characteristics of its own network to maximize the ability of comparison. The discriminator model uses this ability to identify the type of the time–frequency samples and give the judgment result, that is, synthetic feature samples or real feature samples. The generator and discriminator continuously conduct bilateral game alternating training, and finally achieve the purpose of expanding the time–frequency samples of high-quality vibration. The training process of the designed DCGAN network of improvement is shown in Fig. 21.

It can be seen from Fig. 21 that the training process of discriminator model D and generator model G can be regarded as a zero sum game. Let *x* = *G*(*z*), feedback the input data to the generator model, and the cost functions of both can be expressed as:

15 $$A^{\left( D \right)} = - \frac{1}{2}E_{{x \sim P_{data} }} \log D\left( x \right) - \frac{1}{2}E_{{x \sim P_{z} }} \log \left( {1 - D\left( {G\left( z \right)} \right)} \right)$$

16 $$A^{\left( G \right)} = \frac{1}{2}E_{{x \sim P_{data} }} \log D\left( x \right){ + }\frac{1}{2}E_{{x \sim P_{z} }} \log \left( {1 - D\left( {G\left( z \right)} \right)} \right)$$

where, *A*^(*D*)^ is the cost function of *D*;* A*^(*G*)^ is the cost function of *G*; *E*() represents the mathematical expectation of solving data; *P*_*data*_ is the original data input to improve DCGAN network; *P*_*z*_ is the random noise data of improved DCGAN network input.

Set up:

17 $$V\left( {\varsigma^{\left( D \right)} ,\varsigma^{\left( G \right)} } \right) = E_{{x \sim P_{data} }} \log D\left( x \right) + E_{{x \sim P_{z} }} \log \left( {1 - D\left( {G\left( z \right)} \right)} \right)$$

Then exists:

18 $$\left\{ \begin{gathered} A^{\left( D \right)} = - \frac{1}{2}V\left( {\varsigma^{\left( D \right)} ,\varsigma^{\left( G \right)} } \right) \hfill \\ A^{\left( G \right)} = \frac{1}{2}V\left( {\varsigma^{\left( D \right)} ,\varsigma^{\left( G \right)} } \right) \hfill \\ \end{gathered} \right.$$

In order to realize the game relationship between *D* and *G*, it is necessary to find a solution set of *V*(*ς*^(*D*)^,*ς*^(*G*)^). Finally, the requirement for obtaining *D* is the maximum, and the requirement for obtaining *G* is the minimum. Therefore, in the network training process, set the likelihood function and solve its maximum optimization value:

19 $$\begin{gathered} S = \prod\limits_{i = 1}^{m} {P_{G} } \left( {x^{i} ;\varsigma } \right) \hfill \\ \varsigma^{*} { = }\arg \mathop {\max }\limits_{\varsigma } \prod\limits_{i = 1}^{m} {P_{g} \left( {x^{i} ;\varsigma } \right)} = \arg \mathop {\max }\limits_{\varsigma } \log \prod\limits_{i = 1}^{m} {P_{g} \left( {x^{i} ;\varsigma } \right)} \approx \arg \mathop {\max }\limits_{\varsigma } E_{{x \sim P_{data} }} \left[ {\log P_{g} \left( {x^{i} ;\varsigma } \right)} \right] \hfill \\ \begin{array}{*{20}c} {} \\ \end{array} \begin{array}{*{20}c} {} \\ \end{array} = \arg \mathop {\max }\limits_{\varsigma } \int\limits_{x} {P_{data} } \log P_{g} \left( {x^{i} ;\varsigma } \right)dx = \arg \mathop {\max }\limits_{\varsigma } \left[ {\int\limits_{x} {P_{data} } \log P_{g} \left( {x^{i} ;\varsigma } \right)dx - \int\limits_{x} {P_{data} } \log P_{data} \left( {x^{i} ;\varsigma } \right)dx} \right] \hfill \\ \end{gathered}$$

where, *m* represents the number of samples in *Batch* during training.

Let *G* be fixed and unchanging, and use the maximum optimization value solution algorithm of Eq. (19) to reorganize Eq. (17) into integral form, then the optimal solution model of *D* can be expressed as:

20 $$V_{f\left( x \right)} = \int {P_{data} } \left( x \right)\log D\left( x \right) + P_{g} \left( x \right)\log \left( {1 - D\left( x \right)} \right)dx$$

21 $$\frac{{dV_{f\left( x \right)} }}{dD\left( x \right)} = \frac{{P_{data} \left( x \right)}}{D\left( x \right)} = \frac{{P_{g} \left( x \right)}}{1 - D\left( x \right)} = 0$$

22 $$D^{*} \left( x \right) = \frac{{P_{data} \left( x \right)}}{{P_{data} \left( x \right) + P_{g} \left( x \right)}}$$

where, D* represents the optimal solution model of discriminator; *P*_*g*_ is the data synthesized by the generator. When the characteristics between the synthesized data *P*_*g*_(*x*) and the original data *P*_*data*_(*x*) are approximated, the discriminator outputs approximately 1/2 of the data results.

If D*(*x*) is substituted into Eq. (19), there is:

23 $$V\left( {G,D^{*} } \right) = \int {P_{data} } \left( x \right)\log \frac{{P_{data} \left( x \right)}}{{P_{data} \left( x \right) + P_{g} \left( x \right)}}dx + \int {P_{g} \left( x \right)} \log \frac{{P_{g} \left( x \right)}}{{P_{data} \left( x \right) + P_{g} \left( x \right)}}dx$$

The Jensen Shannon divergence probability distribution theorem^31^ is used to transform Eq. (23) to obtain:

24 $$\begin{gathered} V\left( {G,D^{*} } \right) = - \log \left( 4 \right) + KL\left( {P_{data} \parallel \frac{{P_{data} + P_{g} }}{2}} \right) + KL\left( {P_{g} \parallel \frac{{P_{data} + P_{g} }}{2}} \right) = - \log \left( 4 \right) + 2 \times JSD\left( {P_{data} \parallel P_{g} } \right) \hfill \\ = - \log \left( 4 \right) + 2\left( {\int {P_{data} \left( x \right)\log \frac{{P_{data} \left( x \right)}}{{\frac{{P_{data} \left( x \right) + P_{g} \left( x \right)}}{2}}}dx + \int {P_{g} \left( x \right)\log \frac{{P_{g} \left( x \right)}}{{\frac{{P_{data} \left( x \right) + P_{g} \left( x \right)}}{2}}}dx} } } \right) \hfill \\ \end{gathered}$$

where, when *P*_*data*_ = *P*_*g*_, *V*(*G*,D*) takes the minimum value, − log (4), and the generator model G* reaches the optimal state.

### Result analysis

#### Generation of the time–frequency synthesized sample image in the coal and rock cutting state

The improved DCGAN model was used to train the samples of the coal and rock cutting state types. Parameter settings are shown in Table 7.

**Table 7:** Improved parameter setting of DCGAN sample augmentation model.

Parameter	Numerical value
Learning rate	0.002
Optimization strategy	Adam
Batch size	128
Loss function	Cross entropy
Number of pre-generated samples of each type	5000

**Figure 22:**
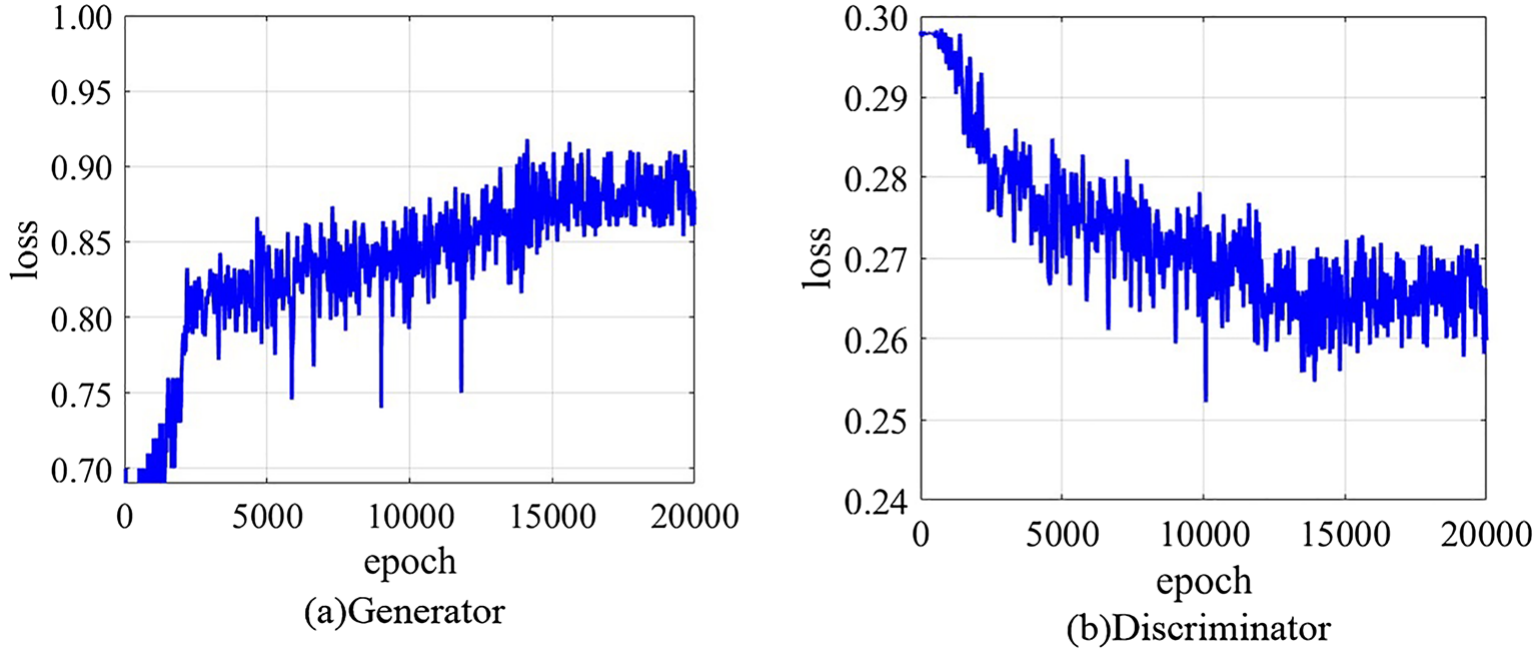
The change relationship between the number of iterations and the training loss.

After repeated experiments and tests, the output results show that when the number of iterations of the training process is set to 20,000, the improved DCGAN network model reaches the most ideal training state, and the change relationship between the number of iterations and the training loss is shown in Fig. 22.

It can be seen from Fig. 22 that the generator model changes steadily in the initial stage of the training process, and then the loss value shows an upward trend with the increase of the number of iteration rounds. When the number of training iterations reached 5867, 6634, 9008 and 11,811 in the process of rising change, there was a shock with large amplitude change. When the number of iterations is 14,549, the loss value gradually tends to a gentle state, and there is no large up and down change of oscillation amplitude thereafter, which indicates that the confrontation between the generator model and the discriminator model is over. The change trend of the loss value of the discriminator model is opposite to that of the generator model. The loss value shows a downward trend, and there is an obvious large-scale fluctuation in the process of downward change, which indicates that the discriminator model has not yet found the direction of the best solution. The model is still in the learning period, and there is still confrontation training between the generator and the discriminator. After 14,546 rounds of iterative training, the discriminator model approaches the direction of the optimal solution, and the loss value is stable without large amplitude oscillation. This shows that the similarity between the synthetic vibration time–frequency sample image and the real original vibration time–frequency image has reached a very close state, and the quality of the synthetic sample image is at the highest level.

**Figure 23:**
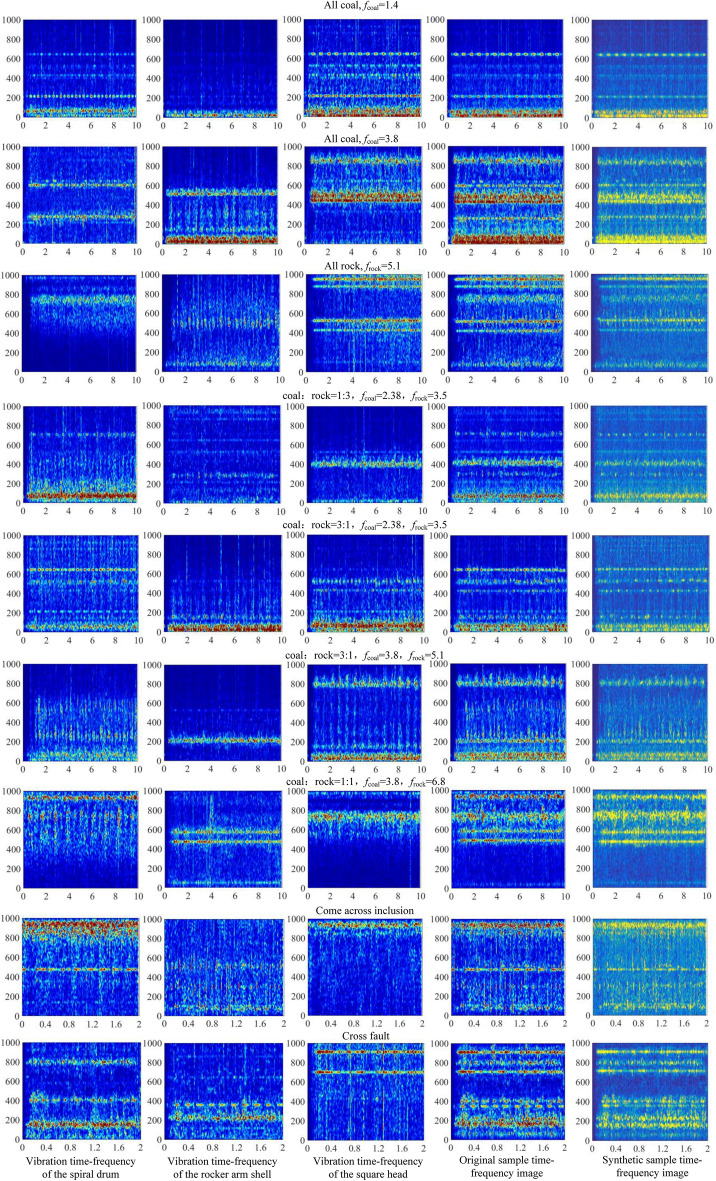
Synthetic sample.

We structured the time–frequency synthesized sample image of the coal and rock cutting state by improved DCGAN network. Due to article length limitation, only 9 working conditions were randomly extracted in Fig. 23 for display.

It can be seen from the comparison between the synthetic image and the real image in Fig. 23 that the image synthesized by the improved DCGAN network highly simulates the characteristics of the real samples obtained in the experiment. The similarity between them is very high, but their details have significant differences. This shows that the designed model not only enriches the database of the coal and rock cutting status, but also achieves the goal of generating high-quality images that can represent different coal and rock cutting status.

#### Comparative analysis of different models

**Figure 24:**
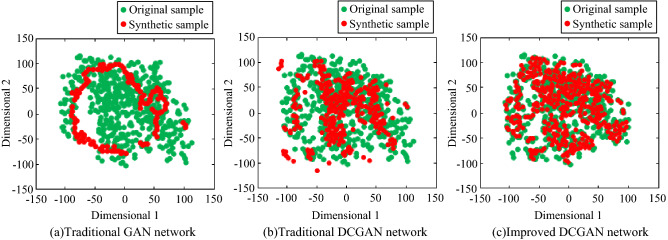
Comparison of image distribution of synthetic time–frequency samples under different network models.

In order to further verify the superiority of improving the performance of DCGAN network, traditional GAN network and traditional DCGAN network were used to expand the spectrum image samples. During the experiment, the training parameters of the traditional GAN network and the traditional DCGAN network were consistent with the improved DCGAN network, and they were trained and learned respectively until they reach the convergence state. The samples synthesized by the three algorithms implement transparent visual display after dimension reduction, as shown in Fig. 24.

It can be seen from Fig. 24 that the samples synthesized by the traditional GAN network do not all learn the distribution of the real samples, and only a few samples fall in the area where the real samples are located. This is because in order to achieve the convergence effect quickly, the generator synthesizes new samples in the direction of cheating the discriminator samples, resulting in the poor diversity of synthesis samples. Compared with the traditional GAN network, the traditional DCGAN network has improved the diversity of sample synthesis effects, and the coverage of synthetic samples in the real sample area has been improved. However, some data still can not learn the distribution characteristics of real samples until the network reaches the convergence state. The improved DCGAN network synthetic sample more accurately find the region where the real samples are distributed. The accuracy of distribution has been significantly improved. It improves the learning ability of the network. The improved DCGAN network tends to quickly synthesize samples with better diversity effects.

## Design of the coal and rock cutting state identification classification model based on RFCNN network

In order to improve the identification accuracy of the coal and rock cutting status, it is necessary to use a identification network with good feature extraction and classification identification effect to train the constructed vibration time–frequency spectrum image dataset. CNN network has significant advantages in image identification. However, during the construction of the coal and rock cutting status identification network, the coal and rock cutting status data has a large amount of information and complex working conditions. Using traditional CNN network model to classify and identify them will lead to problems such as too long training period and slow identification and classification speed. Therefore, this paper combined the advantages of fast convergence of classifier in machine learning algorithm with CNN network. The classifier of CNN network model was optimized by using random forest classification decision. According to the characteristics of the time–frequency spectrum image of the vibration that represents the coal and rock cutting state, a identification model of the coal and rock cutting state was designed, which had rapid convergence, high identification accuracy and short classification identification period. In order to facilitate the description of the network in this paper, the network model of the coal and rock cutting status identification was marked as the RFCNN network model.

### Structure design of RFCNN network model

**Figure 25:**
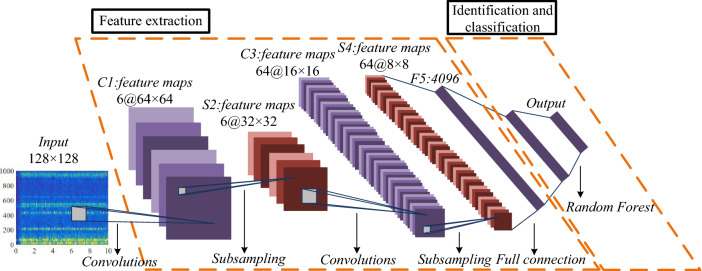
RFCNN network structure model.

The designed RFCNN network model consists of two main structures, namely, the feature extraction layer of CNN network and the identification and classification layer of Random Forest classifier. The specific RFCNN network structure model is shown in Fig. 25.

**Figure 26:**
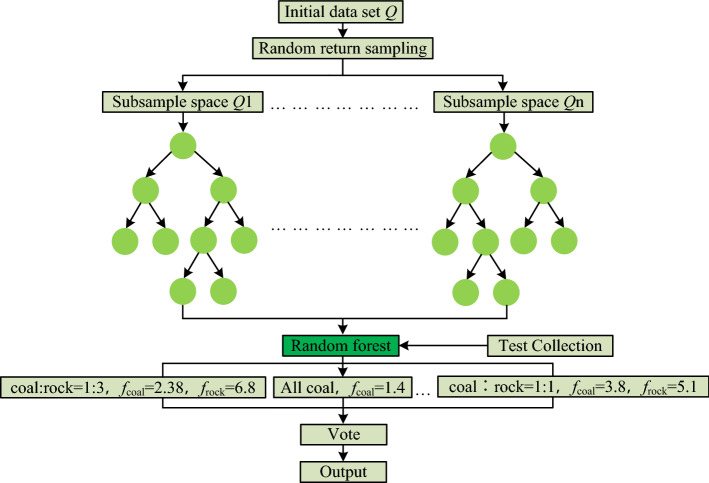
Identification and classification process of Random Forest identification and classification layer.

It can be seen from Fig. 25 that the RFCNN network model, based on the advantages of CNN network feature extraction, uses two-layer convolution, two-layer maximum pooling and full connection structure to achieve the extraction of vibration time–frequency spectrum sample image features when coal and rock cutting state. The network finally inputs the result feature map obtained from the CNN network feature extraction layer to the Random Forest identification classification layer, and the classifier gives the result of coal and rock cutting state identification. The specific identification and classification process of Random Forest identification and classification layer is shown in Fig. 26.

It can be seen from Fig. 26 that *n* training subspaces can be formed in the process of *n* times of random sampling with releasability in the feature map set *Q*. The C4.5 algorithm^32^ was used to generate the corresponding decision tree from *n* training subspaces. The decision tree will be formed into a random forest for classification and identification of test sets. For the input test set in the random forest, each decision tree will give a classification label, and finally count the classification results of all decision trees for voting, and obtain the final identification results of the RFCNN network model on the coal and rock cutting state according to the law of the majority. In the process of setting the parameters of the Random Forest classifier model, the maximum number of feature maps allowed for each decision tree in the construction process is 7, and the maximum number of decision trees is 500.

### Selection of training parameters

In the training process of RFCNN network model, learning rate and batch size are important super parameters of the network^33,34^. Better learning rate and batch size values can promote the network to obtain better performance. Batch size refers to the number of parameters used for training in the single input network model^35,36^, and the learning rate can be expressed by Eq. (25)^37,38^:

25 $$\alpha_{lr} = \alpha_{c} \times \beta^{{\frac{{i_{lr} }}{{S_{lr} }}}}$$

where, *α*_c_ is the initial learning rate; *β* is the attenuation coefficient; *i*_*lr*_ is the number of iterative training in the current state; *S*_*lr*_ is the time interval of iterative update. Since the initial learning rate in the gradient descent method can usually be set to 0.01, the main parameter affecting the learning rate is the attenuation coefficient. The process of solving the optimal learning rate was transformed into seeking the optimal attenuation coefficient.

**Figure 27:**
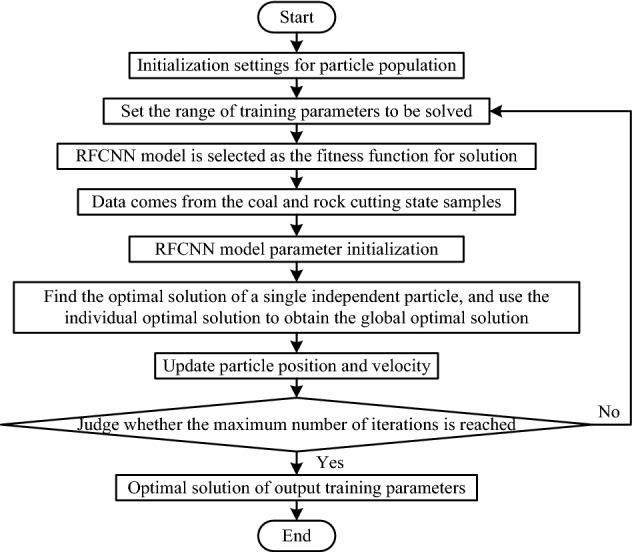
Particle swarm optimization process for solving the optimal training parameters of RFCNN network model.

Based on the optimization criteria of particle swarm optimization algorithm, the optimal values of learning rate and batch size in the RFCNN network model were solved. The process is shown in Fig. 27.

Because the RFCNN network model belongs to a complex nonlinear network, the RFCNN network model acts as a fitness function, which was solved by particle swarm optimization algorithm. First, set the population size of particles to 200, and set the value range of attenuation coefficient and batch size training parameters to the change range of flight speed during particle flight. The set attenuation coefficient and batch size value range are shown in Table 8.

**Table 8:** Value range of attenuation coefficient and batch size.

Attenuation coefficient																				
0.5	0.53		0.55	0.6		0.63	0.65	0.7		0.73	0.75		0.8	0.83	0.85		0.9	0.93		0.95
Batch size																				
20		23			25		27		30			32		35		37			40	

Then, the coal and rock cutting state samples were normalized, and the parameters of the RFCNN network model were initialized. The fitness value of each particle was calculated by RFCNN fitness function, and its optimal position in flight space was recorded. The RFCNN model was used to continuously update the position of a single particle and its own flight speed, obtained the optimal solution of the particle population under the current number of iterations through the optimal solution of each independent particle, and continue to iterate until the optimal solution was output after the maximum number of iterations. The quality of the output optimal solution was judged by the fitness. The accuracy of the final training of the RFCNN network model and the root mean square error of the identification classification were taken as the evaluation criteria for the optimal solution. The solution result of attenuation coefficient is shown in Table 9.

**Table 9:** Solution of attenuation coefficient and experimental results.

Sample type	Attenuation coefficient	Acc	RMSE
The vibration time–frequency spectrum samples of the coal and rock cutting state	0.5	0.7957	0.0694
	0.53	0.8016	0.0727
	0.55	0.8481	0.0662
	0.6	0.7941	0.0651
	0.63	0.8233	0.0691
	0.65	0.7812	0.0715
	0.7	0.8069	0.0681
	0.73	0.8293	0.0656
	0.75	0.8127	0.0678
	0.8	0.8525	0.0693
	0.83	0.8319	0.0592
	0.85	0.8892	0.0644
	0.9	0.9014	0.0539
	0.93	0.8764	0.0574
	0.95	0.8611	0.0564

**Figure 28:**
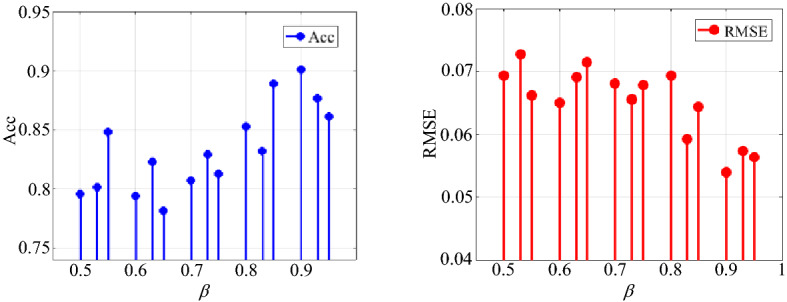
Fluctuation of Acc and RMSE with different attenuation coefficients.

It can be seen from Table 9 that the corresponding Acc and RMSE vary widely under different attenuation coefficients. The result data in Table 9 shows that the evaluation criteria fluctuate with the change of attenuation coefficient, as shown in Fig. 28. It can be seen from Fig. 28 that when the attenuation coefficient is at the 13th level, Acc is the maximum and RMSE is the minimum. Therefore, when the attenuation coefficient is 0.9, both the accuracy rate and the root mean square error evaluation criteria are optimal. Finally, the optimal value of the attenuation coefficient of the RFCNN network model trained by the vibration time–frequency spectrum samples in the coal and rock cutting state is determined to be 0.9.

After the optimal value of the attenuation coefficient of the RFCNN network model was determined, the same particle swarm optimization algorithm was used to find the optimal value of the batch size. Set the initial learning rate of the RFCNN network model to 0.01, the number of iterations to 5000, and the attenuation coefficient to the optimal value of 0.9. The experimental results of solving the batch size are shown in Table 10.

**Table 10:** Experimental results of batch size solution.

Sample type	Batch size	Acc	RMSE
The vibration time–frequency spectrum samples of the coal and rock cutting state	20	0.8922	0.0595
	23	0.8734	0.0556
	25	0.9177	0.0524
	27	0.8963	0.0587
	30	0.8749	0.0605
	32	0.8912	0.0591
	35	0.8671	0.0613
	37	0.8828	0.0657
	40	0.8591	0.0630

**Figure 29:**
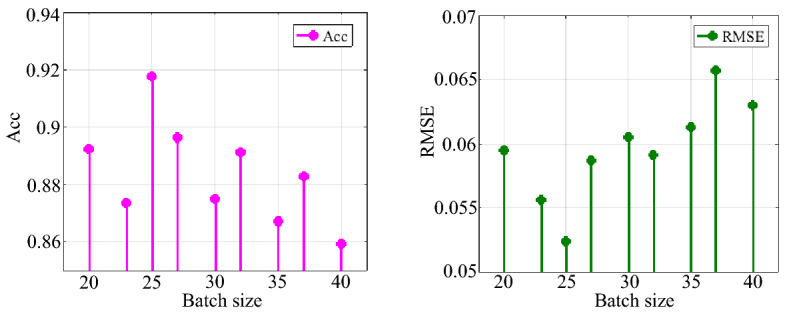
Fluctuation of Acc and RMSE with different batch size.

It can be seen from Table 10 that, compared with the experimental results of attenuation coefficient, the change of batch size results in a relatively narrow range of ACC and RMSE. The result data in Table 10 shows the fluctuation of the evaluation criteria with the change of batch size, as shown in Fig. 29. It can be seen from Fig. 29 that when the batch size is at the third level, the evaluation effect of the evaluation criteria reaches the best state, that is, the accuracy is the highest, and the root mean square error is the lowest. Finally, the optimal value of the batch size of the RFCNN network model trained by the vibration time–frequency spectrum samples in the coal and rock cutting state is determined to be 25.

### Comparative verification and analysis of models

#### Comparative analysis of different recognition network models

**Figure 30:**
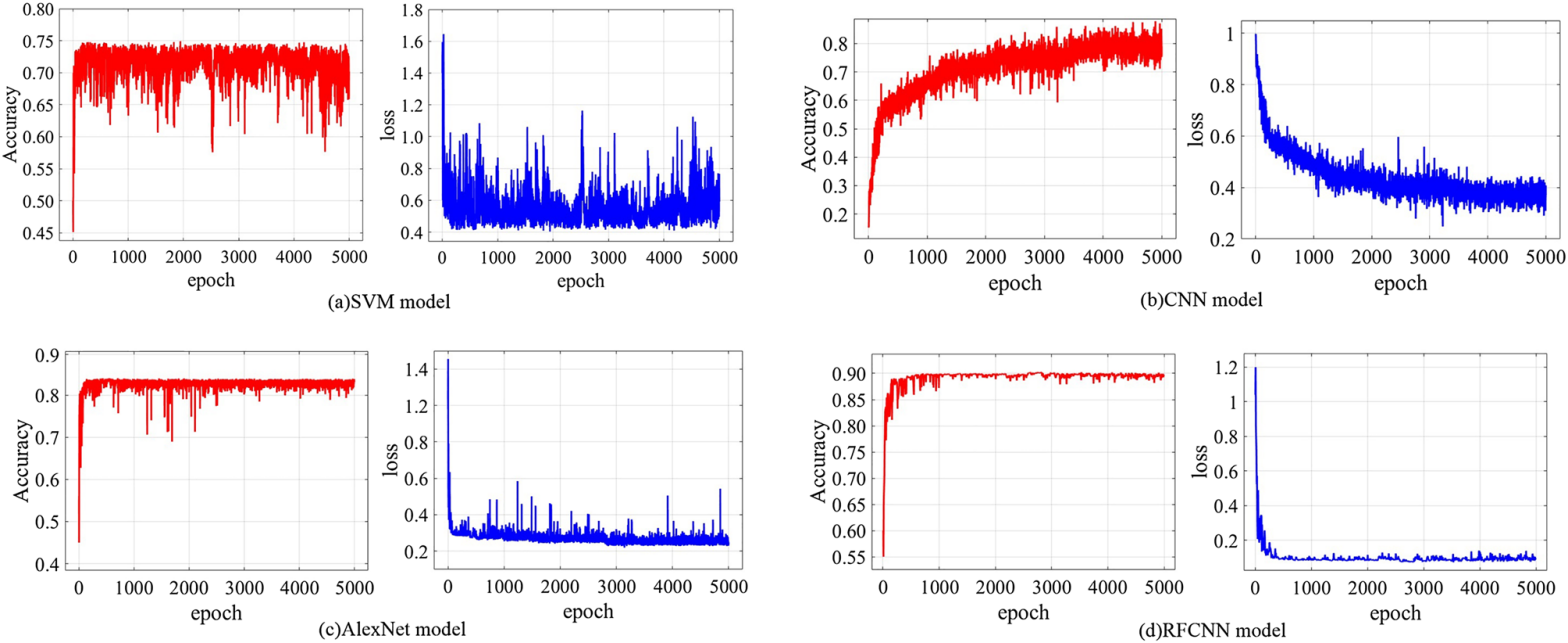
Recognition accuracy and loss function of model.

The commonly used support vector machine (SVM) model, CNN model and Alexnet model were selected to design a comparative experiment with RFCNN network model. The setting parameters of each network model were consistent. The number of input samples for each type of working condition was set to 256, with 5000 iterations. The results were extracted after the test, as shown in Fig. 30. It can be seen from Fig. 30 that with the increase of the number of iterations, the recognition accuracy of the four different network models increases accordingly. When the number of iterations reaches a certain value, the recognition accuracy increases slowly and the network tends to converge. Through comparison, it can be seen that RFCNN network has the fastest convergence speed and the highest recognition accuracy.

In order to further verify the generalization ability and stability of RFCNN network, each model was tested repeatedly for 5 times, and the test results are shown in Table 11. It can be seen from Table 11 that the average recognition accuracy of RFCNN network model is the highest, reaching 91.011%, which is 25.805%, 12.958 and 9.326% higher than that of SVM, CNN and AlexNe respectively. Meanwhile, the standard deviation of RFCNN network model is 25.781, 21.925 and 19.337% lower than that of SVM, CNN and AlexNe respectively. This shows that RFCNN network model improves the network performance of the coal and rock cutting state recognition, ameliorates the stability of model training, and makes the network recognition system have better generalization performance. In addition, the variance and average deviation of the recognition accuracy of RFCNN network model are the smallest. It can be seen that RFCNN network model has significant advantages in fitting performance compared with the other three network models.

**Table 11:** Performance comparison of different recognition network models.

Network model name	Average value of recognition accuracy/%	Standard deviation of recognition accuracy	Variance of recognition accuracy	Average deviation of recognition accuracy
RFCNN	91.011%	1.022 × 10^−3^	1.114 × 10^−6^	0.0419
AlexNet	83.247%	1.267 × 10^−3^	1.646 × 10^−6^	0.0891
CNN	80.571%	1.309 × 10^−3^	1.712 × 10^−6^	0.1531
SVM	72.343%	1.377 × 10^−3^	2.057 × 10^−6^	0.2032

#### Comparative analysis of the number of synthetic samples on the recognition effect of Rfcnn network model

In order to verify the influence of using the improved DCGAN model to expand samples on the training and learning of RFCNN model, different numbers of synthetic samples were generated respectively. The original samples and synthetic samples were mixed as the training set and test set of RFCNN network. After repeated tests for 5 times, the indicators to measure the recognition accuracy were calculated, and the results are shown in Table 12. It can be seen from Table 12 that when the number of synthetic samples added is 0, the average recognition rate of RFCNN network is 88.641%. When the number of synthetic samples added is 5000, the average recognition rate reaches 98.344%, which is 10.946% higher than that of the first group of models. The variance and average deviation of the recognition accuracy are the smallest, but when the number of synthetic samples exceeds 5000, the average recognition rate changes little. It shows that the improved DCGAN network can improve the recognition ability of the coal and rock cutting state of RFCNN network. For the DCGAN-RFCNN network designed in this paper, when the number of synthetic samples reaches 5000, the recognition effect is the best. At the same time, with the increase of the number of synthetic samples, the standard deviation of the recognition rate of the model first decreases and then increases. When the number of synthetic samples is 5000, although the average recognition rate is only 0.859% higher than that of the model with the number of synthetic samples of 4000, the standard deviation of the recognition rate is reduced by 85.729%. It shows that the effective expansion of data not only improves the accuracy of the coal and rock cutting state recognition network, but also improves its generalization ability.

**Table 12:** Statistics of various indicators of recognition accuracy of RFCNN network model under different number of synthetic samples.

Experimental grouping	Number of synthetic samples	Average value of recognition accuracy/%	Standard deviation of recognition accuracy	Variance of recognition accuracy	Average deviation of recognition accuracy
1	0	90.641	1.226 × 10^−3^	1.357 × 10^−6^	6.736 × 10^−2^
2	1000	93.262	3.258 × 10^−4^	1.205 × 10^−7^	3.284 × 10^−3^
3	2000	94.447	2.042 × 10^−4^	5.329 × 10^−8^	2.538 × 10^−4^
4	3000	96.193	1.577 × 10^−4^	1.969 × 10^−8^	2.303 × 10^−4^
5	4000	97.506	1.201 × 10^−4^	1.374 × 10^−8^	1.627 × 10^−4^
6	5000	98.344	1.714 × 10^−5^	2.883 × 10^−10^	1.115 × 10^−5^
7	6000	98.307	1.722 × 10^−5^	2.917 × 10^−10^	1.132 × 10^−5^
8	7000	98.295	1.718 × 10^−5^	2.904 × 10^−10^	1.127 × 10^−5^

## Experimental verification

**Figure 31:**
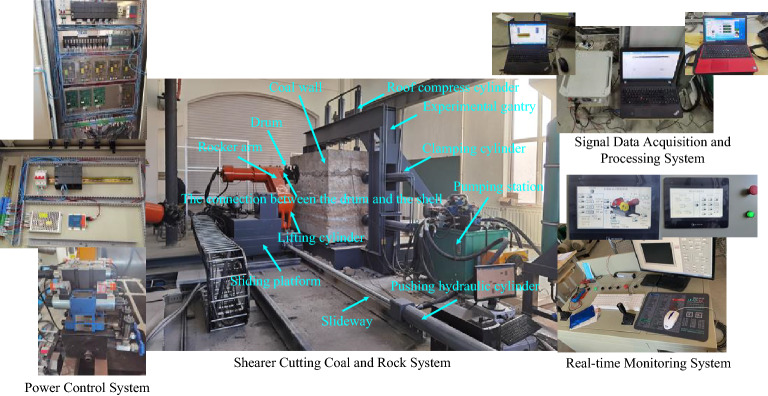
Experimental platform for shearer cutting coal and rock.

In order to verify the superiority of the network model in the identification of the coal and rock cutting status, experimental research was carried out in the Liaoning Provincial Key Laboratory of Large-Scale Mining Equipment^39,40^. Based on the original mining equipment in the laboratory, the existing experimental platform for the coal and rock cutting by shearers was reconstructed, which is mainly composed of four parts: the coal and rock cutting system, signal data acquisition and processing system, control power system and real-time monitoring system, as shown in Fig. 31.

**Figure 32:**
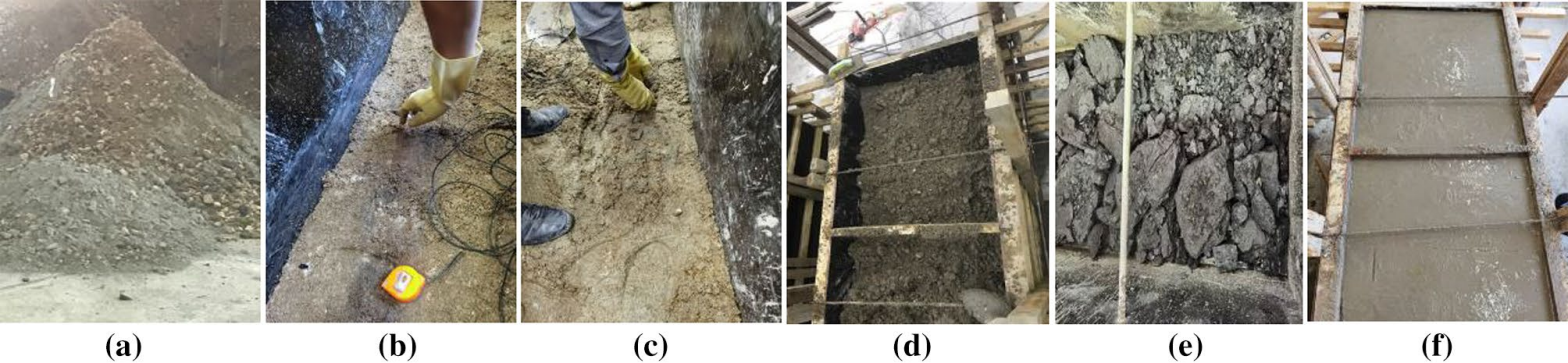
Artificial simulation of coal wall making process.

The artificial simulated coal wall is based on the gangue containing coal and rock in 4602 working face of Yangcun Mine of Yanzhou mining area. According to the similarity ratio, the prototype numerical results corresponding to the experimental coal wall are calculated, as shown in Table 13. By comparing the numerical results of the similar model and the prototype, the errors of the prototype bonding parameters and the results of the similar model after back extrapolation are within the allowable range^41–43^, both less than 3.5%. This verifies the correctness of the deduction of the similarity criterion of the bonding parameters, that is, the method of making the coal wall based on the similarity theory is feasible, and the specific process of making it is shown in Fig. 32.

**Table 13:** Comparison and verification of prototype and artificial coal wall model parameters.

Parameters	Prototype	Artificial coal wall	Similar reverse result	Error
Normal stiffness of coal ~ coal $k_{n} /\left( {N \cdot m^{ - 3} } \right)$	1.1098E + 08	5.5566E + 07	1.0853E + 08	2.21%
Normal stiffness of coal ~ rock $k_{n} /\left( {N \cdot m^{ - 3} } \right)$	1.4158E + 08	7.2277E + 07	1.4116E + 08	0.30%
Normal stiffness of roal ~ rock $k_{n} /\left( {N \cdot m^{ - 3} } \right)$	1.9548E + 08	9.9224E + 07	1.9380E + 08	0.86%
Tangential stiffness of coal ~ coal $k_{s} /\left( {N \cdot m^{ - 3} } \right)$	8.5104E + 07	4.3052E + 07	8.4097E + 07	1.18%
Tangential stiffness of coal ~ roal $k_{s} /\left( {N \cdot m^{ - 3} } \right)$	1.0857E + 08	5.4668E + 07	1.0678E + 08	1.65%
Tangential stiffness of roal ~ roal $k_{s} /\left( {N \cdot m^{ - 3} } \right)$	1.4989E + 08	7.6096E + 07	1.4863E + 08	0.84%
Normal stress of coal ~ coal $\sigma /pa$	8.0017E + 06	4.0458E + 06	7.9022E + 06	1.24%
Normal stress of coal ~ roal $\sigma /pa$	1.6356E + 07	8.0872E + 06	1.5795E + 07	3.43%
Normal stress of roal ~ roal $\sigma /pa$	2.5375E + 07	1.2867E + 07	2.5131E + 07	0.96%
Tangential stress of coal ~ coal $\tau /pa$	2.2232E + 06	1.1215E + 06	2.1905E + 06	1.47%
Tangential stress of coal ~ roal $\tau /pa$	7.0743E + 06	3.5924E + 06	7.0161E + 06	0.82%
Tangential stress of roal ~ roal $\tau /pa$	1.2539E + 07	6.2426E + 06	1.2193E + 07	2.76%

**Figure 33:**
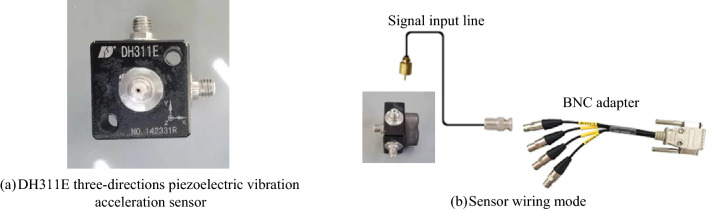
DH311E three-directions piezoelectric vibration acceleration sensor.

**Figure 34:**
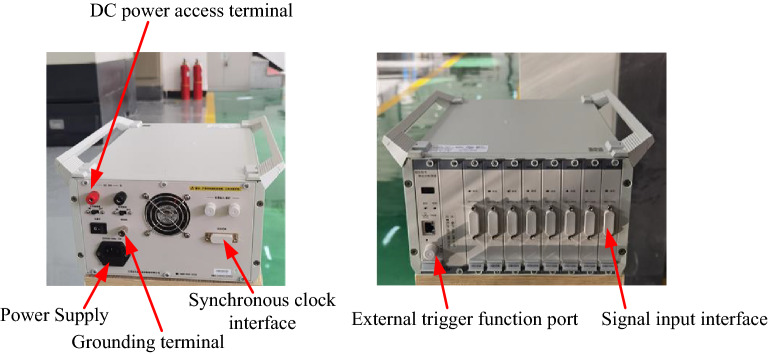
DH5922D signal test analyzer.

The signal data acquisition and processing system mainly includes vibration acceleration sensor, signal test analyzer and data storage computer. The vibration acceleration sensor used in the experiment adopts DH311E three-directions piezoelectric vibration acceleration sensor, as shown in Fig. 33. The model of signal test analyzer is DH5922D, as shown in Fig. 34. After the vibration acceleration sensor was processed by the signal test analyzer, the data information can be transmitted to the data storage computer through Ethernet communication. At the same time, DHDAS software platform was installed inside the data storage computer to analyze and process the data signals transmitted by the signal test analyzer simply and conveniently.

**Figure 35:**
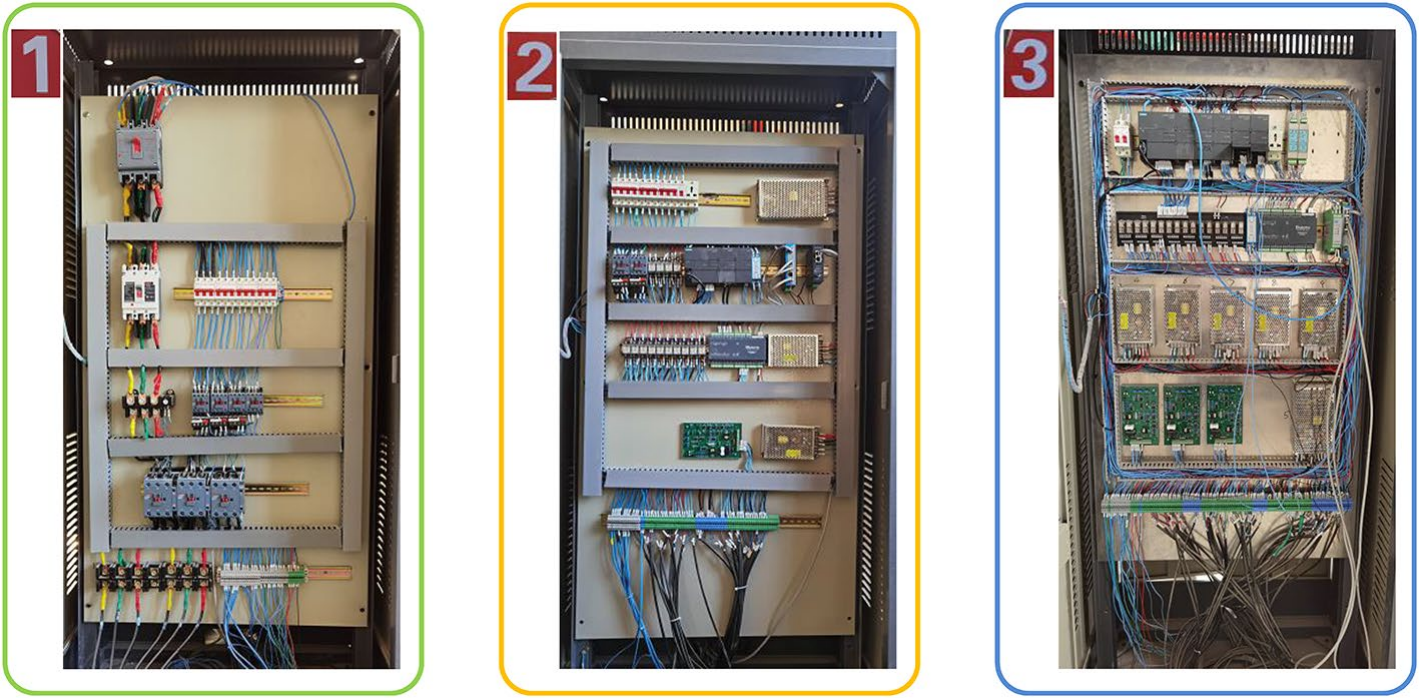
Design and distribution of some hardware structures of control power system.

The control power system mainly realizes the automatic control of the shearer's cutting mechanism, traveling mechanism and coal wall clamping mechanism, including the regulation of the shearer's traction speed and drum rotation speed, the adjustment of the drum height, the reciprocating of the traveling mechanism, the expansion and contraction of the hydraulic cylinder used to clamp the coal wall, and the start and stop of the equipment. The design distribution of some hardware structures of the control power system is shown in Fig. 35.

**Figure 36:**
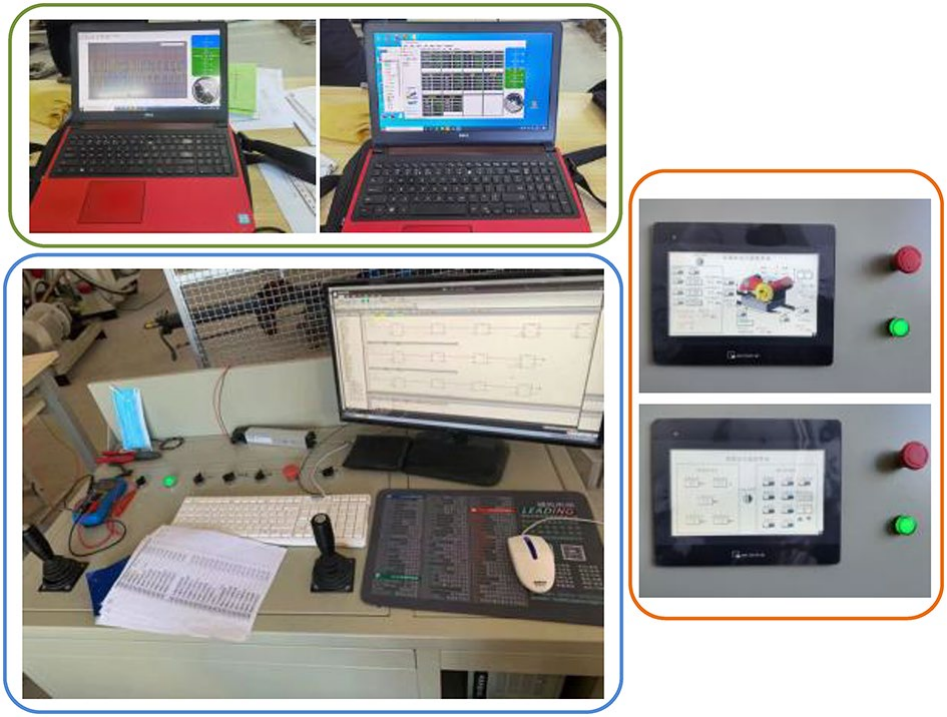
Real time monitoring system.

The real-time monitoring system is mainly composed of coal machine operation monitoring interface and manual monitoring console, as shown in Fig. 36. The coal machine operation monitoring interface is used to monitor the working process of the shearer cutting coal and rock experimental platform. Its operation status and parameter changes of controls can be displayed in real time, so that it can be adjusted and handled in time in case of emergency, and the safety and reliability of the experimental system can be improved. The manual monitoring console consists of a start button, a manual adjustment handle, and a graphical monitoring interface developed based on LabVIEW language. The experimenter can adjust the position and posture of the shearer before the experiment, observe the operation data of the shearer during the experiment, and control the quick retraction of the drum after the experiment through the manual monitoring platform.

**Figure 37:**
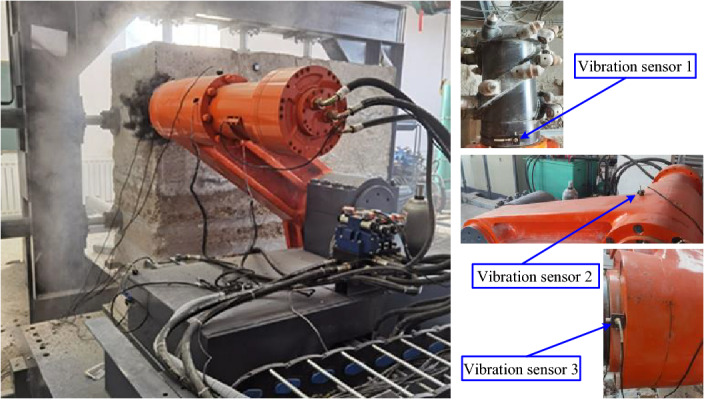
Field test working state.

**Figure 38:**
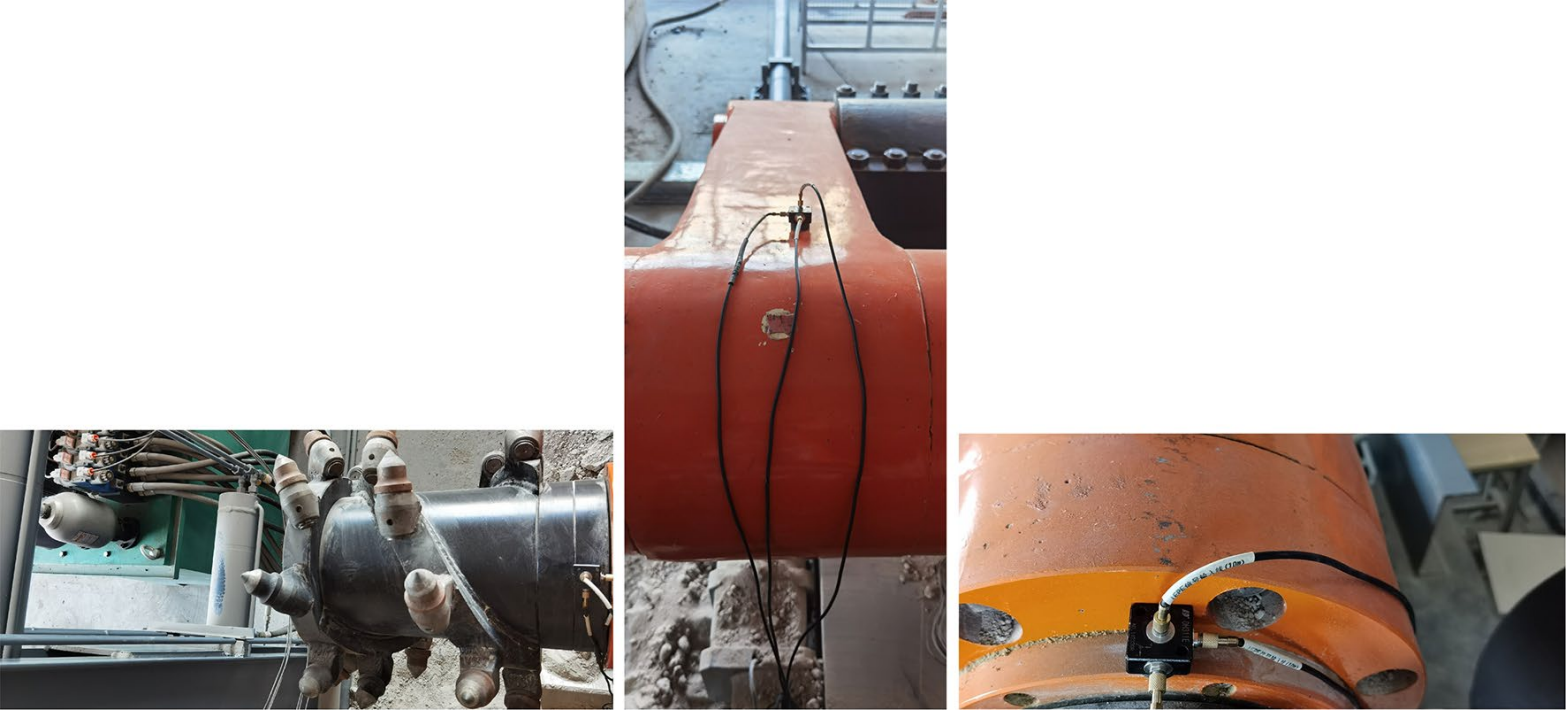
Sensor position after moving.

In this experiment, the coal wall was processed in layers and sections, and six kinds of the coal rock cutting states were set to verify the coal rock cutting state identification system. As shown in Table 14, the cutting experiments were carried out under different working conditions by adjusting the position of the spiral drum. The different structure of the cutting part of the shearer results in different vibration modes, different natural frequencies and different vibration signals. However, the simulation and experimental research based on virtual prototype are consistent with the actual working conditions, and no matter what structural parameters have no effect on the research results. Therefore, in order to maintain the high consistency between the experimental system and the virtual simulation system, DH311E three-directions piezoelectric vibration acceleration sensor was installed at the rear end of the spiral drum of the shearer (vibration sensor 1), the rocker arm shell (vibration sensor 2) and the connection between the drum and the shell (vibration sensor 3). The vibration signals of different parts of the shearer under different cutting conditions were collected through the Signal Data Acquisition and Processing System. The field test working state of this experiment is shown in Fig. 37. Moved the positions of their respective drivers at the rear end of the spiral drum of the shearer, the rocker arm shell and the connection between the drum and the shell relative to the original position in Fig. 37, as shown in Fig. 38. Under the condition of working condition 1 in Table 14, the vibration acceleration measured by the original position sensor and the vibration acceleration of the after moving sensor were extracted for comparison and analysis, and the results are shown in Table 15. From the statistical results in Table 15, it can be seen that the vibration acceleration measured by the sensor after moving the position is similar to that measured by the sensor at its original position. This shows that changing the positions of sensors at the rear end of spiral drum, rocker arm shell and the connection between drum and shell has no influence on the research results.

**Table 14:** Experimental conditions.

Working condition	Type of working condition
1	All coal, *f*_coal_ = 2.38
2	All coal, *f*_coal_ = 3.8
3	Coal:rock = 3:1, *f*_coal_ = 2.38, *f*_rock_ = 3.5
4	Coal:rock = 3:1, *f*_coal_ = 2.38, *f*_rock_ = 5.1
5	Coal:rock = 1:1, *f*_coal_ = 2.38, *f*_rock_ = 6.8
6	Coal:rock = 1:1, *f*_coal_ = 2.38, *f*_rock_ = 7.4

**Table 15:** Comparison and analysis of sensors at different positions.

	The vibration acceleration of original position sensor / mm/s^2^	The vibration acceleration of the after moving sensor / mm/s^2^	Differ /%
At the rear end of the spiral drum of the shearer	5281.319	5176.949	1.976
The rocker arm shell	2683.499	2746.684	2.354
The connection between the drum and the shell	7725.102	7807.186	1.063

**Figure 39:**
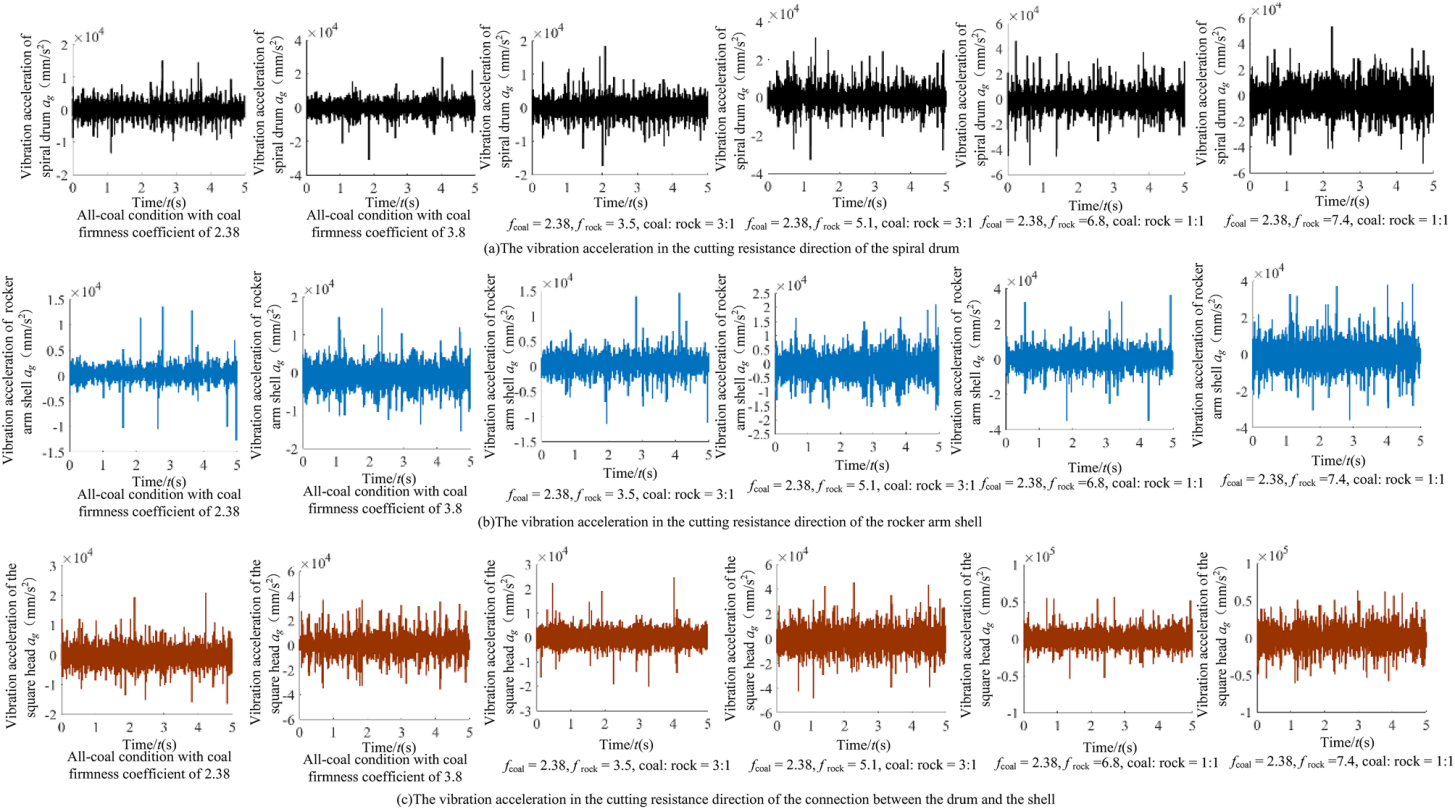
The vibration acceleration in the cutting resistance direction.

**Figure 40:**
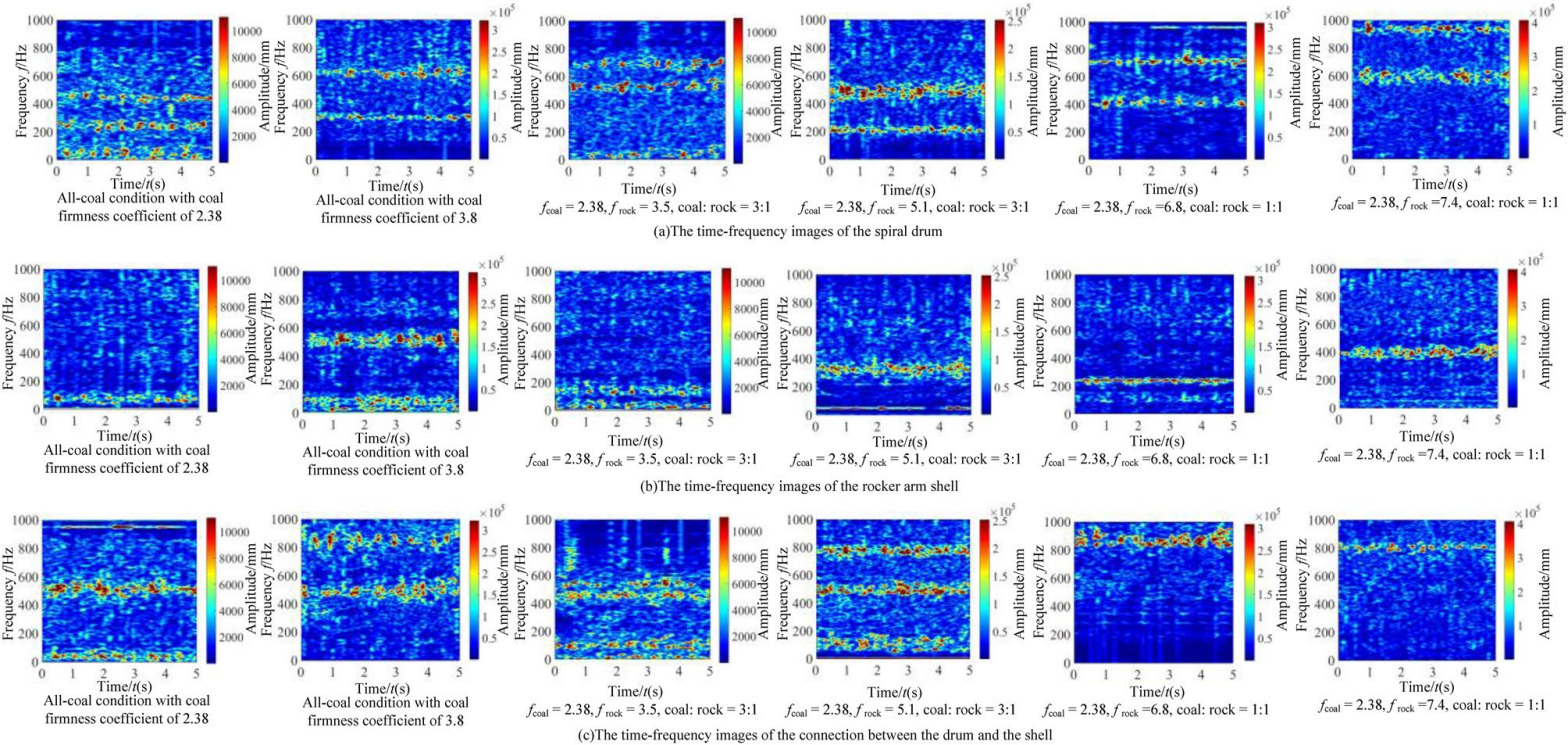
The time–frequency images.

**Figure 41:**
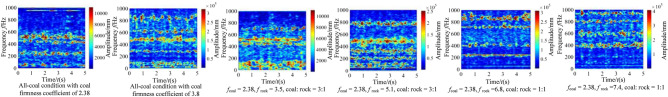
Fused two-dimensional vibration time-spectrum image.

**Figure 42:**
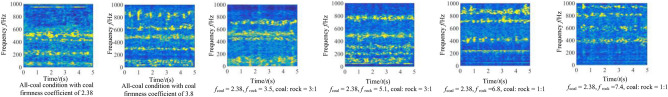
Augmented images of experimental samples.

After the experiment, the vibration signals of spiral drum, rocker arm shell and the connection between drum and shell collected by DH311E three-directions piezoelectric vibration acceleration sensor were drawn as one-dimensional time domain diagram by DH5922D signal test analyzer. Among them, the vibration signals of the coal and rock cutting state under each different working conditions were divided into 28 groups of sample data information with a duration of 5S. The one dimensional time domain data samples were transformed into the two-dimensional time–frequency images according to the STFT data information conversion method constructed in this paper. Then, the designed MW coal and rock cutting vibration feature fusion rules were used to fuse the feature information of the two-dimensional vibration time–frequency images of the spiral drum, the rocker arm shell and the connection between the drum and the rocker arm shell. The fused original samples used the improved DCGAN network model to generate synthetic samples, with 5000 iterations. A part of experimental samples are shown in the Figs. 39, 40, 41 and 42. Finally, the “big data” includes 5000 images under each working condition. After the training of the improved DCGAN network model was completed, based on the migration learning, the obtained synthetic samples were mixed with the original samples, the training set and test set were divided by 4:1, and then input into the RFCNN network to identify the cutting state of coal and rock. The results are shown in Table 16.

**Table 16:** Experimental result.

Working condition	1	2	3	4	5	6	Correct sample	Total	Recognition accuracy
1	989	6	2	0	0	0	989	997	99.19%
2	10	998	3	0	0	0	998	1011	98.71%
3	2	3	992	7	5	0	992	1009	98.32%
4	1	1	6	987	3	0	987	998	98.89%
5	0	0	5	3	995	6	995	1009	98.61%
6	0	0	2	5	12	991	991	1010	98.12%
Total sample	—	—	—	—	—	—	5952	6034	98.64%

It can be seen from Table 16 that the recognition accuracy of 6 different coal and rock cutting conditions in the experimental design is more than 95%. Taking working condition 3 as an example, the total number of test samples is 1009, the number of samples correctly identified by the model is 992, and 17 data samples are misjudged. This is because the texture feature of the background domain of the synthetic image causes slight interference to the discrimination results, but this interference accounts for only 1.68% relative to the total samples. Based on the recognition results of the above six different working conditions, the recognition accuracy of the coal and rock cutting state is 98.64%, which has high recognition accuracy and can accurately mapping the coal and rock cutting state. The experimental results verify that using the key technology of the coal and rock cutting state identification constructed in this paper to process the data information can effectively realize the accurate identification of the coal and rock cutting state.

## Conclusion

Under different coal and rock occurrence conditions, the variation difference of vibration information among the spiral drum, rocker arm shell and square head of the shearer is fully preserved in the time–frequency image. There are obvious differences in the location, range and shape of dominant frequency energy between different working conditions.

In the time–frequency image fusion model of MW coal and rock cutting state, the features in the time–frequency image of the spiral drum, the rocker arm shell and the square head vibration information are highly fused and retained. The fused image is used as the basic original data sample of the coal and rock cutting state recognition system, which effectively improves the accuracy of the characteristic sample to represent the coal and rock cutting state and reduces the dependence on a single position sensor.

The results of the extended data set based on the improved DCGAN network show that: There is a high similarity between the samples synthesized by the generator and the original samples, but there are differences between small feature points, which enriches the data set. With the increase of the number of synthetic samples, the recognition rate of the coal and rock cutting state recognition system increases to 98.344%, and then changes slightly. The standard deviation of recognition rate is reduced to 1.714 × 10^−5^, the change is no longer obvious. When the number of synthetic samples is 5000, the recognition effect reaches the best state. By mixing synthetic samples into the data set, the robustness and generalization ability of the coal and rock cutting state recognition model based on deep learning are effectively improved.

Combining the advantages of CNN convolution neural network and Random Forest recognition classifier, the RFCNN coal and rock cutting state recognition network model was designed. The experimental results show that: In the face of complex working conditions such as soft rock-hard coal, more gangue layers and different hardness values of coal and rock, the recognition ability of the coal and rock cutting state of RFCNN network is greatly improved compared with that of ordinary network. Through the laboratory field experiment test, the effective identification of the coal and rock cutting state is realized, and the feasibility of the network is verified.

## Data Availability

All data generated or analysed during this study are included in this published article.
